# TSP-1-CD47-integrin α4β1 axis drives T cell infiltration and synovial inflammation in rheumatoid arthritis

**DOI:** 10.3389/fimmu.2025.1524304

**Published:** 2025-04-16

**Authors:** Jialiang Hu, Xinmin Wang, Chuang Ge, Weiyan Qi, Zeqing Li, Yaoyao Wang, Wenting Lai, Wei Ji, Hanmei Xu

**Affiliations:** ^1^ State Key Laboratory of Natural Medicines, Ministry of Education, China Pharmaceutical University, Nanjing, China; ^2^ The Engineering Research Center of Synthetic Polypeptide Drug Discovery and Evaluation of Jiangsu Province, China Pharmaceutical University, Nanjing, China; ^3^ School of Life Science and Technology, Inner Mongolia University of Science and Technology, Baotou, China; ^4^ Department of Rheumatology and Immunology, Affiliated Hospital of Nanjing University of Traditional Chinese Medicine, Nanjing, China

**Keywords:** rheumatoid arthritis, CD47, thrombospondin-1, integrin α4β1, T cell infiltration, PKA, Src

## Abstract

**Background:**

Immune cell infiltration into joint synovial tissue and promotion of the inflammatory response are important processes in rheumatoid arthritis (RA). This article delves into the crucial role of CD47 in these processes, as well as the mechanisms at both cellular and molecular levels.

**Methods:**

CD47, its ligand TSP-1, and related integrins’ expression was analyzed in RA patients’ synovial and blood samples vs. normals using GEO data. Additionally, a collagen-induced arthritis (CIA) model using *Cd47* knockout rats was employed to explore the significant role of CD47 in the arthritic process. This was further validated in wild-type rat CIA model using CD47 antibodies and inhibitors targeting key enzymes in the CD47-activated integrin α4β1 signaling pathway. The crucial role of CD47 in the CIA model and its way of function were investigated at the animal whole-body level, through various joint section analyses, and at the cellular and molecular level.

**Results:**

Analysis of synovial tissue samples (230 cases) and blood samples (1238 cases) from RA patients in the GEO database showed that the CD47, its ligand TSP-1 and related integrins were significantly overexpressed in RA patients. When *Cd47* was knocked out in a rat CIA model, the disease severity of arthritis was significantly alleviated, and the T cell infiltration into rat synovial tissue was remarkably reduced, while the number of B cells, macrophages, and neutrophils did not noticeably change. Mechanistic studies indicated that CD47 on T cells interacts with TSP-1 on vascular endothelial cells in arthritic synovium, activating T cell integrin α4β1. The activated α4β1 binds to VCAM-1, promoting T cell infiltration and inflammatory factor secretion, thereby exacerbating synovial inflammation. The present study also showed that inhibiting the activities of key kinases PKA and Src, through which CD47 mediated integrin α4β1 activation, alleviated arthritis syndromes in CIA rats.

**Conclusion:**

The three-molecule model of “TSP-1, CD47 and integrin α4β1” confirmed that CD47 plays an important role in the occurrence and progression of collagen-induced arthritis, a typical animal model of rheumatoid arthritis. Blocking the TSP-1-CD47 interaction or inhibiting CD47-activated integrin α4β1 on T cells could be a potential therapeutic strategy for rheumatoid arthritis.

## 1 Introduction

Rheumatoid arthritis is a systemic inflammatory disease, affecting approximately 0.5-1% of the population worldwide (1). The main feature of rheumatoid arthritis is persistent synovial inflammation, which is accompanied by the infiltration of immune cells into the synovium, including neutrophils, lymphocytes, and monocytes (2, 3), these immune cells are activated and produce cytokines that mediate inflammation, which exacerbates the development of synovial inflammation, leading to the formation of pannus and mediating joint damage and bone erosion in the later stages of the disease (4, 5). In 1999, Margereta et al. reported that *Cd47* knockout mice can resist the clinical symptoms of arthritis induced by intravenous injection of S. aureus, and hypothesized that this is due to the slowdown and attenuation of infiltration and activation of monocytes, neutrophils, and T lymphocytes into the joint (6).

CD47 is expressed by virtually all cells in the body (7), which has two ligands: signal-regulatory protein-α (SIRP-α) and TSP-1. SIRP-α is highly expressed on the surface of myeloid cells, and its interaction with CD47 provides a “don’t eat me” signal to macrophages, therefore CD47 antibodies are used in the treatment of hematological malignancies (8–10). Another ligand of CD47 is TSP-1, which interacts with heparin sulfate polysaccharides on the surface of vascular endothelial cells, anchoring to the luminal surface and accumulating on the surface of endothelial cells at sites of inflammation. It can interact with CD47 on the surface of immune cells in the blood (11). CD47 is also known as integrin-associated protein, and it can bind and activate integrins on the same cell surface after interacting with extracellular ligands such as TSP-1. For example, by TSP-1engagement, CD47 can activate integrin αvβ3 on human melanoma cells (12), αIIbβ3 on platelets (13), and α4β1 on sickle red blood cells (14). In 2001, Ticchioni et al. demonstrated that CD47 plays a crucial role in the adhesion of T cells to vascular endothelial cells at inflammatory sites using CD47 wild-type and defective T cells (15). The authors further showed that this process is associated with integrin α4β1/VCAM-1 through blocking antibodies and indicated in the discussion section that CD47 is involved in regulating the high-affinity/avidity state of α4β1 integrins. In 2003, Vallejo et al. showed that the interaction between TSP-1 on the surface of fibroblast-like synoviocytes (FLS) within synovial membrane tissue and CD47 on the surface of T cells further activates T cells (11). It was further inferred that TSP-1 on the surface of vascular endothelial cells may assist T cells in crossing the blood vessel barrier to enter synovial membrane tissue. These early studies, combined with the significant role integrins play in the process of leukocytes crossing the blood vessel barrier to reach inflammatory sites (16), lead to the hypothesis that the interaction between TSP-1, CD47, and integrins may play a vital role in the infiltration of immune cells, especially T cells, across the blood vessel barrier into synovial membrane tissue during the development of rheumatoid arthritis.

In the analysis of clinical samples from the GEO database, we found that CD47, its ligand TSP-1, several integrin subunits (α4, αm, αL, αv, β1, β2, β3) were significantly overexpressed in the synovial tissue of patients with rheumatoid arthritis. Furthermore, there is a correlation between the expression of integrin subunits α4, αm, αL, β1, and β2 and the expression of CD47. Starting from this, we aim to investigate and answer the following questions: Which immune cell surface CD47 interacts with TSP-1 on the vascular wall of arthritis rat synovial tissue? Which integrin is activated by the CD47-TSP-1 interaction, thereby facilitating the infiltration of that immune cell into the synovial tissue? Furthermore, what downstream intracellular signaling pathways are activated by the CD47-TSP-1 interaction to activate that integrin? To address these questions, we have established a collagen-induced arthritis (CIA) model in rats. CIA model was chosen based on the observation of increased expression of CD47, its ligand TSP-1, several integrin subunits (α4, αm, αL, αv, β1, β2, β3) in synovial tissues of CIA rats and the autoimmune nature during the disease induction. *Cd47* knockout rats were included in CIA model here to explore the potential and critical role of CD47 in arthritis development. Complementary validation was performed in wild-type CIA rats using CD47 antibodies and inhibitors of key enzymes in intracellular signaling pathways. Specifically, we identified key enzymes in the intracellular signaling pathway of T cells through which CD47 activates integrin α4β1 to verify whether inhibiting the activity of these key enzymes would affect arthritis development.

## 2 Results

### 2.1 Expression of CD47 and multiple integrins was upregulated in RA

**Figure 1:**
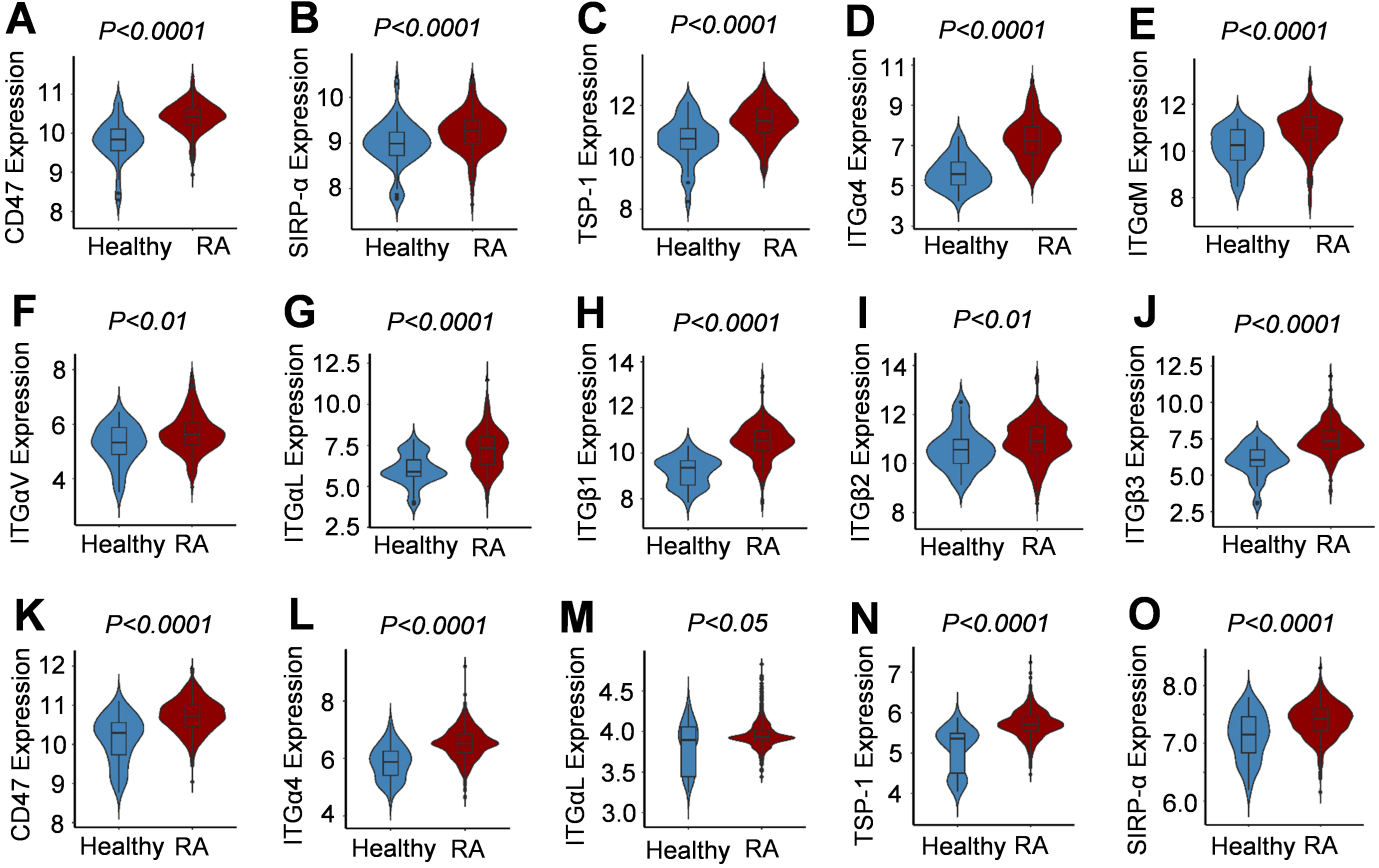
Expression of CD47, its two ligands and several integrin subunits in synovial tissue and peripheral blood samples of human RA patients. **(A–J)** Violin Plots for increased expression of CD47, SIRP-α, TSP-1, integrin subunits (α4, αM, αv, αL, β1, β2, β3) in synovial tissues from RA patients compared with healthy people. Data were based on RA patient synovial tissue samples (RA=230, Normal=59) published on GPL96, GPL570, GPL11154, GPL1708, GPL10558, and GPL91 platforms from NCBI-GEO. **(K–O)**. Violin Plots for increased expression of CD47, SIRP-α, TSP-1, and integrin subunits (α4 and αL) in peripheral blood samples from RA patients compared with healthy people. Data were based on RA patient peripheral blood samples (RA=1238, Normal=120) published on GPL6947, GPL570, GPL20171, and GPL13158 platforms from NCBI-GEO.

By analyzing data from the NCBI-GEO platform, it was found that, based on data from the GPL96, GPL570, GPL11154, GPL1708, GPL10558, and GPL91 platforms, and compared with the synovial samples from healthy people, those from RA patients had significantly higher expression of genes encoding CD47, TSP1, SIRP-α, and integrin subunits α4, αM, αv, αL, β1, β2, and β3 (RA=230, healthy=59) (**Figures 1A–J**). Moreover, based on the data from GPL6947, GPL570, GPL20171, and GPL13158 platforms, the expression of genes encoding CD47, TSP-1, SIRP-α, and integrin subunits α4 and αL was significantly higher in the peripheral blood samples of RA patients than in healthy people (RA=1238, healthy=120) (**Figures 1K–O**). Based on the NCBI/GEO/GSE55457/77298/55235 data, in the synovial tissue samples of RA patients, the expression of genes encoding several integrin subunits (α4, αM, β1, β2, and αL), ICAM-1, and SIRP-α was significantly and positively correlated with CD47 expression (**Supplementary Figure S1**). These results indicated that CD47 may play a pivotal role in RA through related integrin molecules.

### 2.2 Upregulated expressions of CD47 and rheumatoid associated molecules in the synovial tissues of CIA model rats

Collagen-induced arthritis model in wild-type (WT) rats was established, which included two animal groups: the normal group and the model group (WT-CIA) (**Supplementary Figure S2**). Testing from day 13 of modeling found that the rats in the model group had markedly increased swelling of left and right hind paws, and higher arthritis and clinical scores compared with the normal group (**Supplementary Figures S2A–E**). On day 28, the animal blood samples were collected for testing, which revealed that the levels of CIA-associated autoantibodies (including rheumatoid factor, anti-cyclic citrullinated peptide (anti-CCP) antibody, and anti-collagen II (anti-CII) antibody), TNF-α levels, total leukocyte counts, neutrophil counts, and platelet counts were all substantially increased in the blood of the model group compared with the blood samples from rats in the normal group (**Supplementary Figures S2F–L**). These results indicated that rat CIA modeling was successfully set up. We sacrificed the experimental animals on day 28 of modeling, harvested the joint synovial tissues from the hind paws, and performed quantitative real-time polymerase chain reaction (qPCR) and western blot analyses. It was found that in the hind paw joint synovial tissue of the model group animals, the expression of CD47, its two ligands SIRP-α and TSP-1, and integrin subunits α4, αM, β1, and β2 was remarkably higher (**Supplementary Figures S2M–T**). These findings were consistent with the analysis results of the synovial specimens from RA patients, indicating that CD47 may also play an important role in CIA.

### 2.3 *Cd47* knockout alleviated arthritis symptoms in a CIA rat model

**Figure 2:**
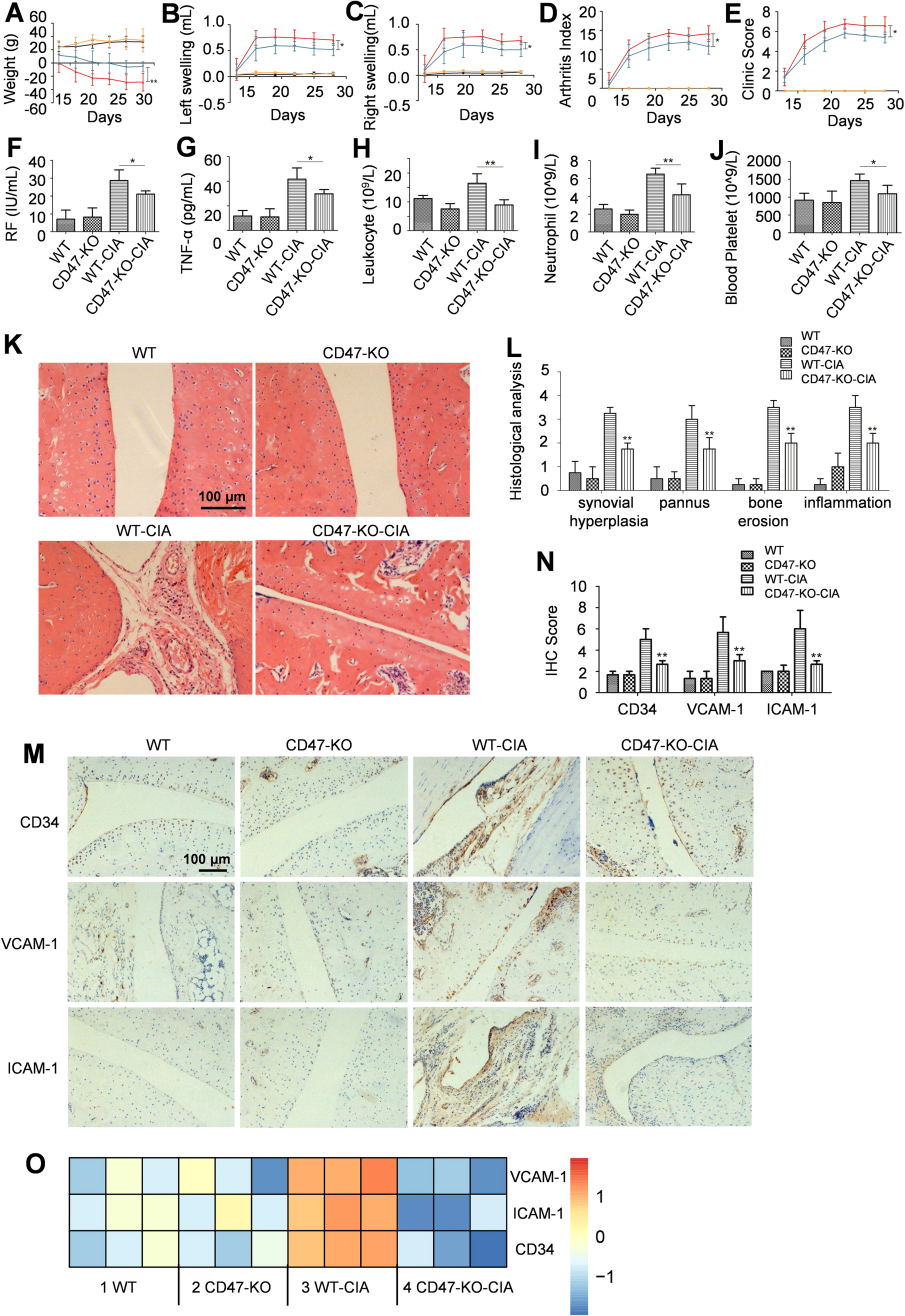
*Cd47* knockout alleviated RA syndromes. Four groups of rats were included (n=5) and for panels **(A–E)** the black line stands for the wild-type rat normal group (WT), the orange line for the *Cd47* knockout rat normal group (CD47-KO), the red line for the wild-type rat model group (WT-CIA), and the blue line for the *Cd47* knockout rat model group (CD47-KO-CIA). **(A–E)** change of rat body weight **(A)**, swelling of hind left paws **(B)** and right paws **(C)** of rats, the arthritis score **(D)** and clinic score **(E)** of rats during the experiment. On day 28 rat blood was collected and the levels of molecules or cells were measured and compared which included rheumatoid factor **(F)**, TNF-α **(G)**, total leukocytes **(H)**, neutrophils **(I)** and platelets **(J)**. For **(A–J)** n=5. All of the data show mean ± SD. *P < 0.05, **P < 0.01. Hind paw joints were taken on day 28 for H&E and immunostaining analysis. **(K)** Representative images of hind paw histology were shown for rats in the four experimental groups (200×) and the presence of hyperplasia of the synovium, pannus formation, inflammation and bone erosion in the joint sections of wild-type rat model group can be seen. **(L)** Histopathologic score analysis confirmed that the extent of arthritis was significantly reduced in the joints in *Cd47* knockout rat model group compared with wild-type rat model group (n=3, three photos for each experimental group). **(M, N)**. Representative images of immunostaining of ankle joint for CD34, ICAM-1, and VCAM-1 (200×) were shown **(M)** and the degree of CD34, VCAM-1, and ICAM-1 staining for the four groups were compared based on analysis with ImageJ 1.51K software (n=3, three photos for each experimental group) **(N)**. For **(L, N)** data show means ± SD. *p < 0.05, **p < 0.01 versus the wild-type rat model group. **(O)** Differential expression of VCAM-1, ICAM-1, and CD34 in synovial membranes of rats in the four groups by RNAseq analysis (n=3, samples of three animals for each group). Differentially expressed genes (for selected relevant molecules) were used to generate gene expression heatmap by Cluster 3.0 with the hierarchical method and R 4.0.3 with the heatmap method.

CRISPR/Cas9 technology was employed in this study to delete the 31 bp target site of the second exon of the *Cd47* gene in Sprague Dawley (SD) rats. After breeding and screening, the homozygous *Cd47* knockout rats were derived (**Supplementary Figure S3**). The systematic protein expression analysis of the tail tissue of a wild-type and a *Cd47* knockout rat revealed that CD47 was minimally expressed in the *Cd47* knockout rat, while the expression of other related molecules did not differ between the wild-type and *Cd47*-deficient rats (**Supplementary Figure S3D**). We utilized wild-type rats and *Cd47* knockout rats to establish the CIA models, which included four groups of animals: the wild-type rat normal group (WT), the *Cd47* knockout rat normal group (CD47-KO), the wild-type rat model group (WT-CIA), and the *Cd47* knockout rat model group (CD47-KO-CIA) (**Figure 2**). Compared with the WT-CIA group, the CD47-KO-CIA group exhibited significant decreases in the swelling of the left and right hind paws, arthritis and clinical scores, as well as levels of rheumatoid factor (RF), TNF-α, total leukocytes, neutrophils, and platelets in the blood (**Figures 2A–J**). There was no significant difference in levels of anti-CCP and anti-collagen II antibodies between WT-CIA and CD47-KO-CIA groups.

On day 28 of modeling, the hind paw joints from animals were collected to perform histopathological analysis. Compared with the WT normal group rats, severe synovial tissue hyperplasia, pannus formation, inflammation, and bone erosion could be seen on the hind paw ankle joint sections of animals in the WT-CIA group, whereas on the hind paw ankle joint sections of the CD47-KO-CIA group, these symptoms were alleviated (**Figures 2K, L**). Immunohistochemical analysis of ankle joint sections for CD34, VCAM-1, and ICAM-1 expression revealed there was less vascular endothelial cell proliferation of the ankle joint synovial tissue in the CD47-KO-CIA group than in the WT-CIA group animals (**Figures 2M, N**). RNAseq analysis results of the synovial tissue in the experimental animals also demonstrated that compared with the WT-CIA group, the CD47-KO-CIA group had reduced expression of these vascular marker molecules in the ankle joint synovial tissue as well (**Figure 2O**).

**Figure 3:**
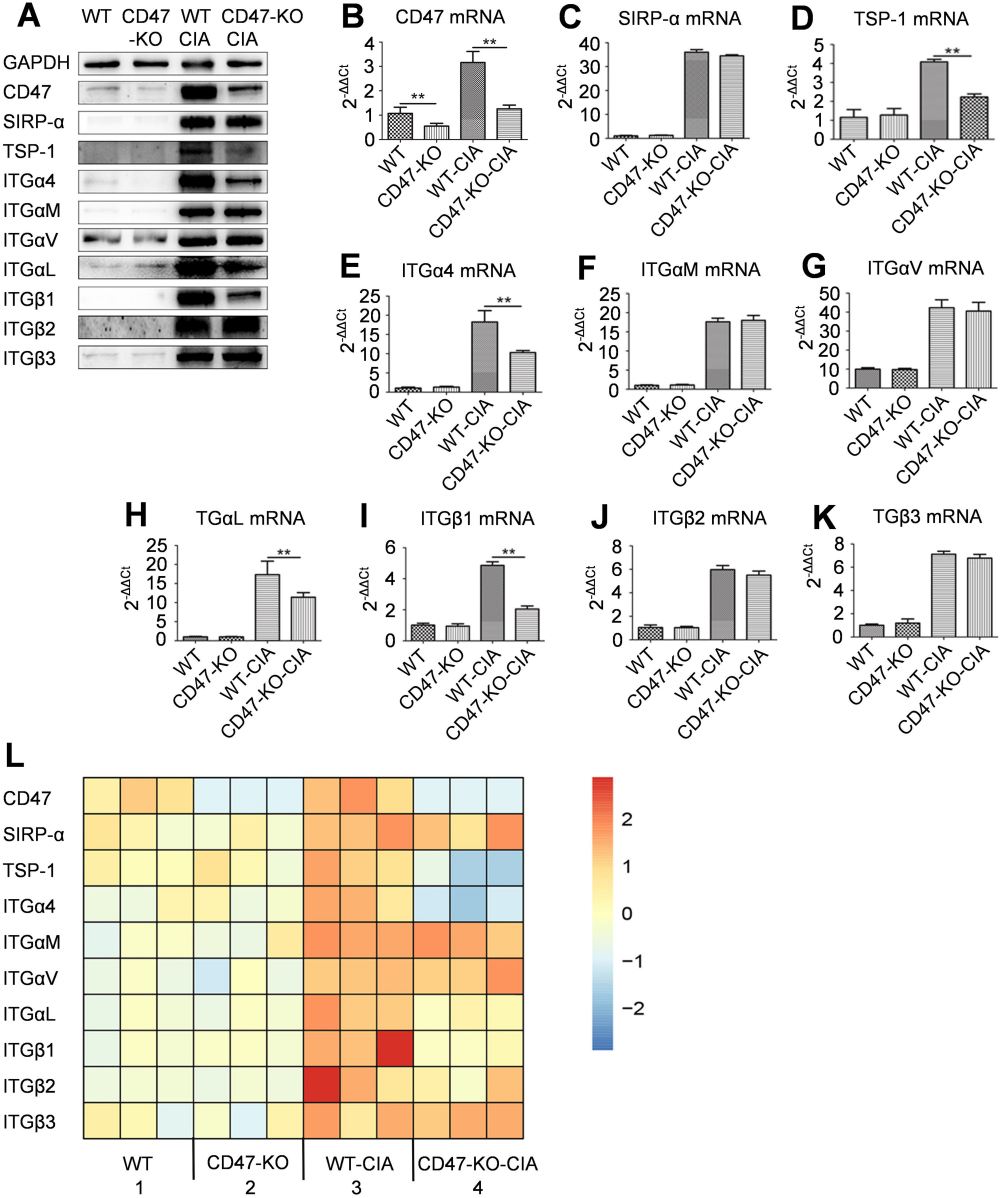
Expression of CD47, its two ligands and several integrin subunits in rat synovial tissues. On day 28 of the animal experiment shown in **Figure 2**, rats were sacrificed and synovial tissues of the hind paws were taken for western-blot, quantitative real-time PCR, and RNAseq analysis. **(A)** Photo of western-blot for the expression of CD47, SIRP-α, TSP-1, integrin subunit α4, αM, αv, αL, β1, β2 and β3. **(B–K)** Expression of CD47 **(B)**, SIRP-α **(C)**, TSP-1 **(D)**, integrin subunit α4 **(E)**, αM **(F)**, αv **(G)**, αL **(H)**, β1 **(I)**, β2 **(J)** and β3 **(K)** on an mRNA level in synovial membranes of rats in the four groups were shown and compared. For **(B–K)**, n=3 (three replicates for detection of each molecule). All of the data show mean ± SD. **P < 0.01. **(L)** Differential expression of CD47, its two ligands (SIRP-α and TSP-1), and several integrin subunits in synovial membranes of rats in the four groups by RNAseq analysis (n=3, samples of three animals for each group). Gene expression heatmap was generated by Cluster 3.0 with the hierarchical method and R 4.0.3 with the heatmap method.

Hind paw synovial tissue of animals was also collected on day 28 of modeling to perform western blot and qPCR analyses. Compared with the WT normal rats, the WT-CIA animals had significantly elevated expression of CD47, several integrin subunits, SIRP-α, and TSP-1 in the hind paw synovial tissue. In comparison with animals in the WT-CIA group, the hind paw synovial tissue expression of CD47, TSP-1, and integrin subunits α4, αL, and β1 decreased in the CD47-KO-CIA group animals, while there were no changes in the expression of SIRP-α and integrin subunits αM, αv, β2, and β3 (**Figures 3A–K**).

RNAseq analysis on the synovial tissue of the experimental animals from each group was used to find that compared with the animals of the WT-CIA group, those of the CD47-KO-CIA group had decreased expression of the genes encoding TSP-1 and integrin subunits α4, αL, and β1 in synovial tissue, while the expression of genes encoding SIRP-α and of the integrin subunits αM, αv, β2, and β3 were not altered (**Figure 3L**).

The above experiments analyzed the overall clinical appearance of the animals, tested for key factors and cells in blood, and analyzed experimental animal joint tissue slices, all of which suggested that *Cd47* deficiency significantly reduced the disease severity of model rats, synovial inflammation levels, and vessel hyperplasia. Molecular analysis of the experimental animals’ articular synovial tissue indicated that *Cd47* knockout significantly decreased the expression of TSP-1 and integrin subunits α4, αL, and β1 in the synovial tissue of model animals.

### 2.4 *Cd47* knockout reduced the infiltration of T cells in rat synovial tissue

**Figure 4:**
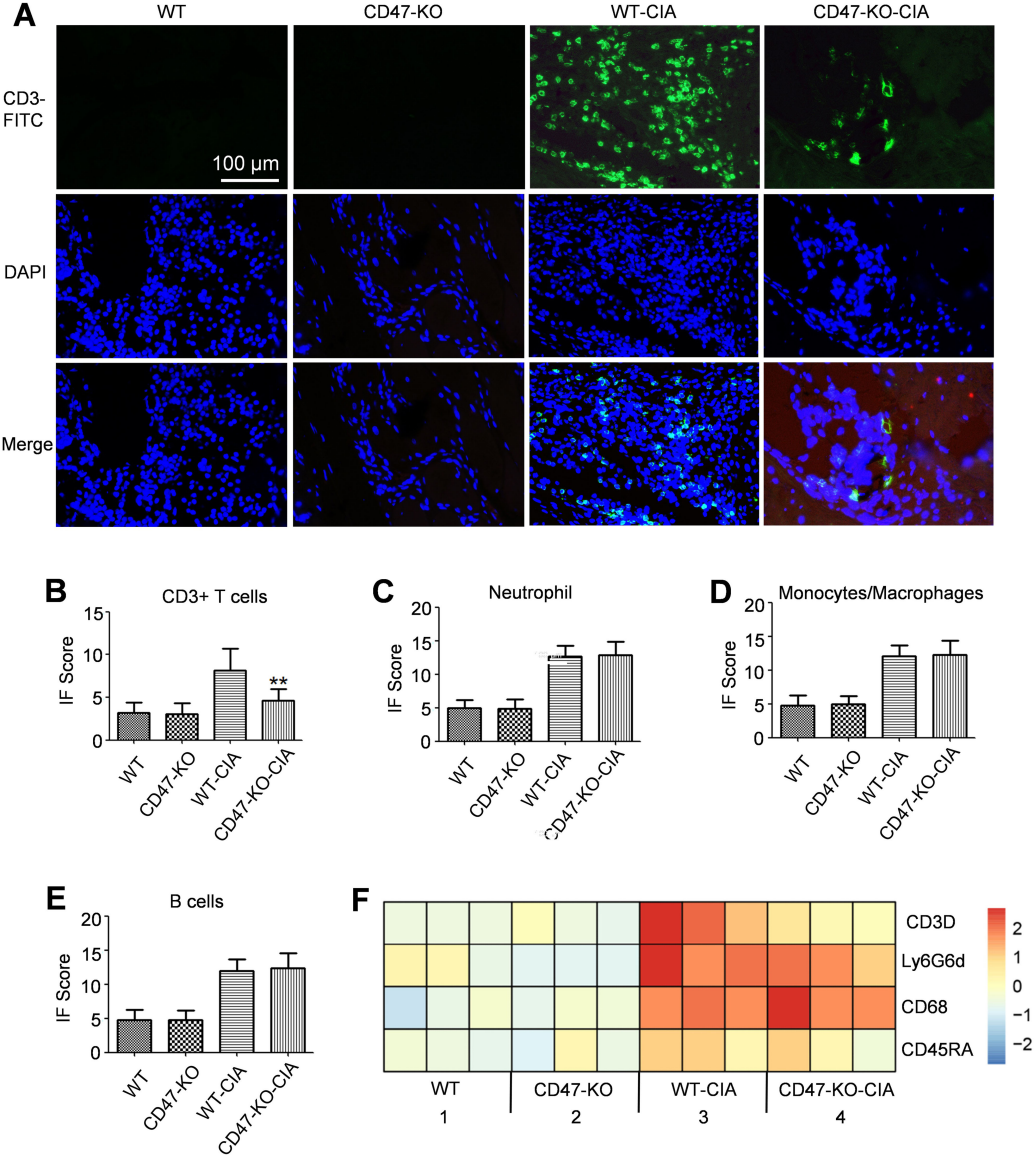
Decreased infiltration of T cells in the synovial membrane of *Cd47* knockout rat model group. Hind paw joints were taken from rats in the animal experiment as shown in **Figure 2** on day 28 and used as samples for immunofluorescence staining analysis. **(A)** Immunofluorescence staining of CD3 expression on hind paw sections (400×). The CD3 molecule was labeled with FITC. DAPI was used to stain the cell nucleus. **(B)** Fluorescence signal analysis confirmed that significantly fewer CD3+ T cells infiltrated the joints of *Cd47* knockout rat model group compared with wild-type rat model group (n=3, three photos for each experimental group). **(C–E)**. Fluorescence signal analysis confirmed that there was no significant difference in the number of neutrophils **(C)**, macrophages **(D)**, and B cells **(E)** infiltrated the joints of rats between the wild-type rat model group and *Cd47* knockout rat model group (n=3, three photos for each experimental group) based on the detection of Ly6G-FITC, CD68-FITC or CD45RA-Cy3 signal individually. For **(B–E)** data show means ± SD. **p < 0.01. **(F)** Differential expression of markers for T cells (CD3D), neutrophils (Ly6G6d), macrophages (CD68), and B cells (CD45RA) in synovial membranes of rats in the four groups by RNAseq analysis (n=3, samples of three animals for each group).

Immunofluorescence analyses were performed on sections of the experimental animals’ hind paw joints for a T cell marker (CD3), a neutrophil marker (Ly6G), a macrophage marker (CD68), and a B cell marker (CD45RA). It was found that compared with the WT-CIA group, the CD47-KO-CIA group had significantly reduced CD3 expression, whereas there were no obvious changes in Ly6G, CD68, or CD45RA expression (**Figures 4A–E**). RNAseq analysis on the joint synovial tissue of experimental animals confirmed the above conclusion at the mRNA level (**Figure 4F**).

Immunofluorescence analysis was further performed for a T cell marker (CD3), a CD4+ T cell marker (CD4), a CD8+ T cell marker (CD8), a Th17 cell marker (IL-23R), and a Treg cell marker (CD25) in the experimental animal joint tissue. It was found that compared with the wild-type rat normal group, the WT-CIA group exhibited significantly increased expression of these markers in the joint tissue sections. In contrast to the WT-CIA group, the CD47-KO-CIA group showed significantly reduced expression of these markers on the joint tissue sections (**Supplementary Figure S4**). We performed RNAseq analysis on the synovial tissue of the experimental animals and found that the change in gene expression encoding marker molecules for various T cell subgroups (CD3D, CD4, CD8a, IL-17A, and FoxP3) agreed with the immunofluorescence analysis results (**Supplementary Figure S4F**). In addition, western blot analysis was further performed for the expression of relevant marker molecules for CD4+T cells, Th17 cells, and Treg cells in the experimental animal synovial tissue, which revealed that in comparison to the WT-CIA group, the CD47-KO-CIA group had substantially decreased expression of these marker molecules in synovial tissues (**Supplementary Figures S4G–I**).

Data shown in **Figure 4** and **Supplementary Figure S4** demonstrated that after modeling of *Cd47*-deficient rats, the number of T cells and several T cell subgroups, including Th17 cells, which infiltrated into joint synovial tissues markedly declined. According to reports in literature, in a CIA model, deficiency of IL-17A (17) or blocking its function by IL-17 antibody (18) considerably attenuated the development of disease symptoms. Thus, *Cd47* knockout could mitigate the severity of arthritis by reducing the infiltration of T cells (especially Th17 cells) into articular synovial tissues.

### 2.5 Interactions between CD47 and integrin α4β1

In the last two parts of study, it was found that *Cd47* deficiency significantly reduced T cell infiltration into articular synovial tissue, and that the expression of TSP-1, CD47, and integrin subunits α4, αL, and β1 noticeably declined in the synovial tissues of model animals. In this part of study, through the interactions between TSP-1, CD47, and integrin α4β1, the important role of CD47 in the process by which T cells pass across vessel walls and infiltrate into synovial tissues was investigated.

**Figure 5:**
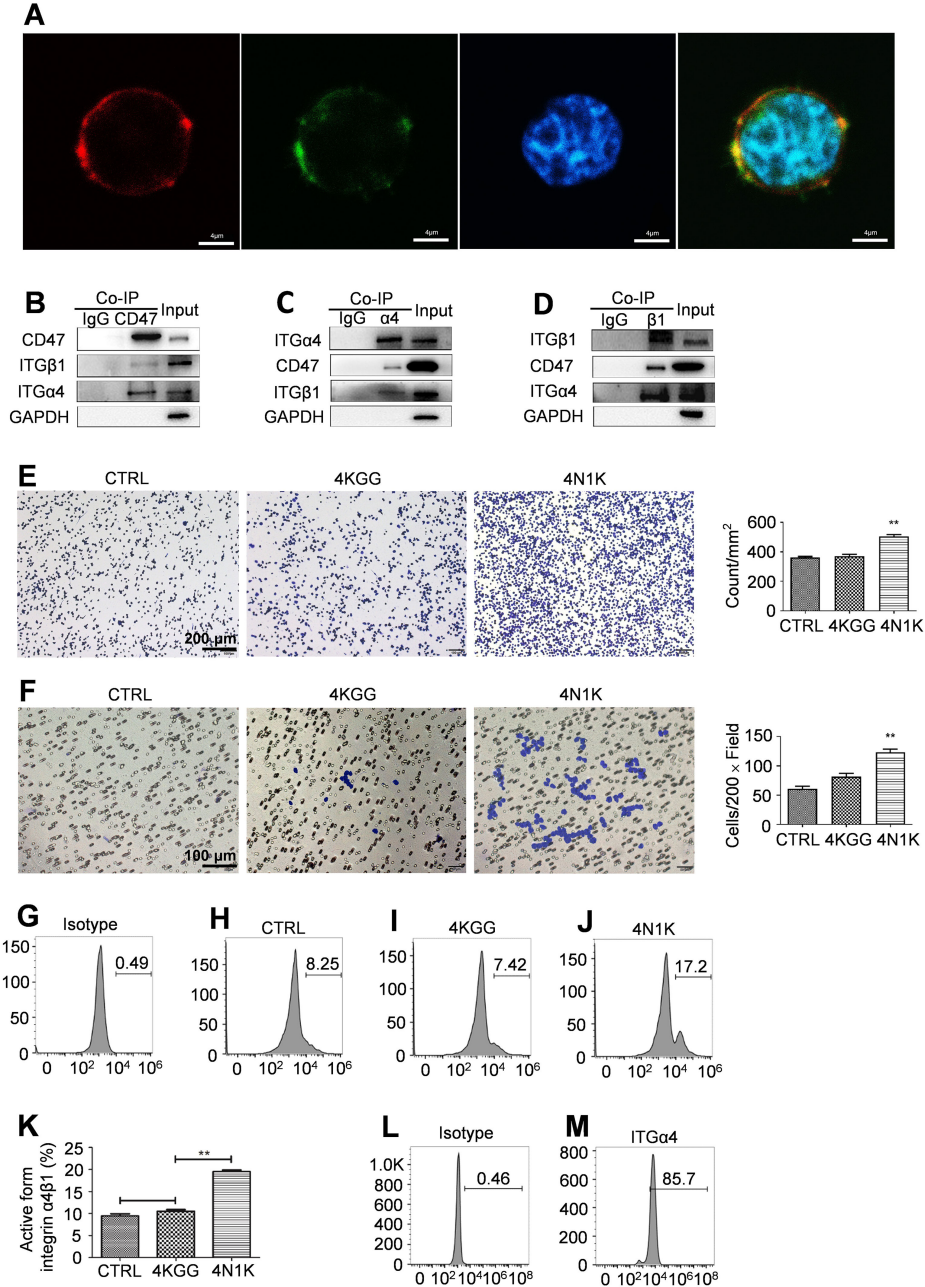
Peptide 4N1K engagement on CD47 increased integrin α4β1 activation and function on human Jurkat cells. **(A)** CD47, α4 integrin, and cell nucleus were probed and stained by an APC conjugated anti-CD47 antibody, a FITC conjugated anti-α4 antibody or Hoechst staining and their merge signal confirmed the interaction between CD47 and α4 integrin on the cell surface (400×). **(B–D)**. Western-blot analysis of the bands for CD47, α4, and β1 integrin subunit after immunoprecipitation with an anti-CD47 **(B)**, anti-α4 **(C)**, or anti-β1 **(D)** antibody. Cell lysate which was negatively labeled on the left was precipitated with an isotype control antibody. The whole-cell lysate on the right was named input. **(E)** Peptide 4N1K treatment increased the adhesion of human Jurkat cells to immobilized VCAM-1 (n=3, three replicate samples for each experimental condition). Cells in the control sample were not incubated with peptides. Typical photos of adhesion cells under various treatment conditions (×100) were shown on the left. **(F)** Peptide 4N1K treatment increased the migration of human Jurkat cells to immobilized VCAM-1 with SDF-1 as a chemoattractant (n=3, three replicate samples for each experimental condition). Typical photos of migrated cells under various treatment conditions (×200) were shown on the left. **(G–J)** Peptide 4N1K increased the percent of “extended form” integrin α4β1 with detection by an anti-active form α4β1 antibody. Cells were treated with peptide 4KGG **(I)**, 4N1K **(J)**, or without peptide treatment **(H)** and then were probed with an anti-active form integrin α4β1 antibody and thereafter a FITC-labeled secondary antibody. Human Jurkat cells incubated with an isotype antibody were used as an isotype control sample **(G)**. Statistical analysis of up-regulation of integrin α4β1 active form was shown in **(K)** (n=3, three detections for the same experimental condition). Data show mean ± SEM. **P < 0.01. Total expression of integrin α4β1 on Jurkat cells was confirmed with the use of a FITC conjugated rabbit anti-human integrin α4β1 monoclonal antibody **(M)**. Jurkat cells incubated with an isotype antibody were used as an isotype control sample **(L)**.

In order to clarify the details of these mechanisms, we first employed laser confocal observations and found that in human T cells (Jurkat cells), the signals from CD47 molecules labeled with red fluorescence and integrin subunit α4 molecules labeled with green fluorescence merged into yellow fluorescence, confirming the interaction between CD47 and integrin α4β1 (**Figure 5A**). Co-immunoprecipitation (co-IP) analysis using CD47, integrin subunit α4, or β1 antibodies also confirmed that CD47 interacted with integrin α4β1 (**Figures 5B–D**).

TSP-1 was continuously present in the synovial tissues of RA patients (19). The peptide 4N1K was derived from TSP-1 and directly participated in the interaction with CD47 (20). Cellular function assays revealed that 4N1K notably promoted Jurkat cell adhesion to immobilized VCAM-1 and migration to immobilized VCAM-1 induced by SDF-1, while the negative control peptide 4KGG did not have these effects (**Figures 5E, F**).

After activation, integrin α4β1 changed from its original folded state to an extended state (15), and the activated integrin α4β1 was detected by an antibody against the extended form of integrin α4β1 in combination with flow cytometry. Peptide 4N1K treatment significantly increased the percentage of the active form of integrin α4β1 on the surface of Jurkat cells (**Figures 5G–K**), while the total cell surface expression of integrin α4β1 remained normal (**Figures 5L, M**).

The above data suggest that by interacting with integrin α4β1, CD47 activated integrin and switched it from the original folded state to the extended state that had a high binding affinity to VCAM-1, enhancing the adhesive and migratory capacity of T cells mediated by VCAM-1.

Integrin αLβ2 is another integrin molecule expressed by T cells, which plays an important role during the special stage when leukocytes pass through the vessel wall into inflamed tissue (21). Through inspection by laser confocal microscopy and co-IP detection, the CD47 on human Jurkat cells was not found to interact with integrin αLβ2 (**Supplementary Figures S5A–D**). Functional assays at the cellular level showed that the 4N1K peptide did not enhance either the adhesion or SDF-1-induced migration of Jurkat cells to immobilized ICAM-1 (**Supplementary Figures S5E, F**). In addition, the proportion of integrin αLβ2 in the active form on Jurkat cells was also not increased after the action of peptide 4N1K on CD47 (**Supplementary Figures S5G–L**). These results indicated that the increased T cell adhesion and migration resulting from the interaction between CD47 and TSP-1 were achieved through the integrin α4β1-VCAM-1 interaction.

The 4N1K peptide significantly increased the wild-type rat CD3+ T cell adhesion to immobilized VCAM-1 (**Supplementary Figure S6A**) and migration induced by SDF-1 (**Supplementary Figure S6C**). The *Cd47*-deficient rat CD3+ T cell adhesion to immobilized VCAM-1 (**Supplementary Figure S6B**) and migration to SDF-1 chemoattractant (**Supplementary Figure S6D**) did not display any differences between the 4N1K treatment group, negative peptide 4KGG treatment group, or normal control cells. Examination by flow cytometry showed that peptide 4N1K significantly increased the amount of the active form of integrin α4β1 on the CD3+ T cell surface of wild-type rats (**Supplementary Figures S6E–K**). In contrast, 4N1K treatment of CD3+ T cells of *Cd47* knockout rats did not noticeably change the quantity of integrin α4β1 in the active form (**Supplementary Figures S6L–O**). These results further verified that the TSP-1 activation of integrin α4β1 on the T cell surface and enhancement of the T cell adhesive and migratory capacity was CD47-dependent.

This set of results explained the critical function exerted by CD47 in T cell infiltration into synovial tissues by establishing that TSP-1-CD47-integrin α4β1 had mutual interactions. The CD47-TSP-1 interaction activated integrin α4β1 on the surface of T cells, and the activated integrin α4β1 had a higher affinity to VCAM-1 on the surface of the vascular endothelial cells, which consequently facilitated T cells to pass through the vascular wall and enter the synovial tissue.

### 2.6 “Inside-out” signaling pathways led to integrin α4β1 activation

**Figure 6:**
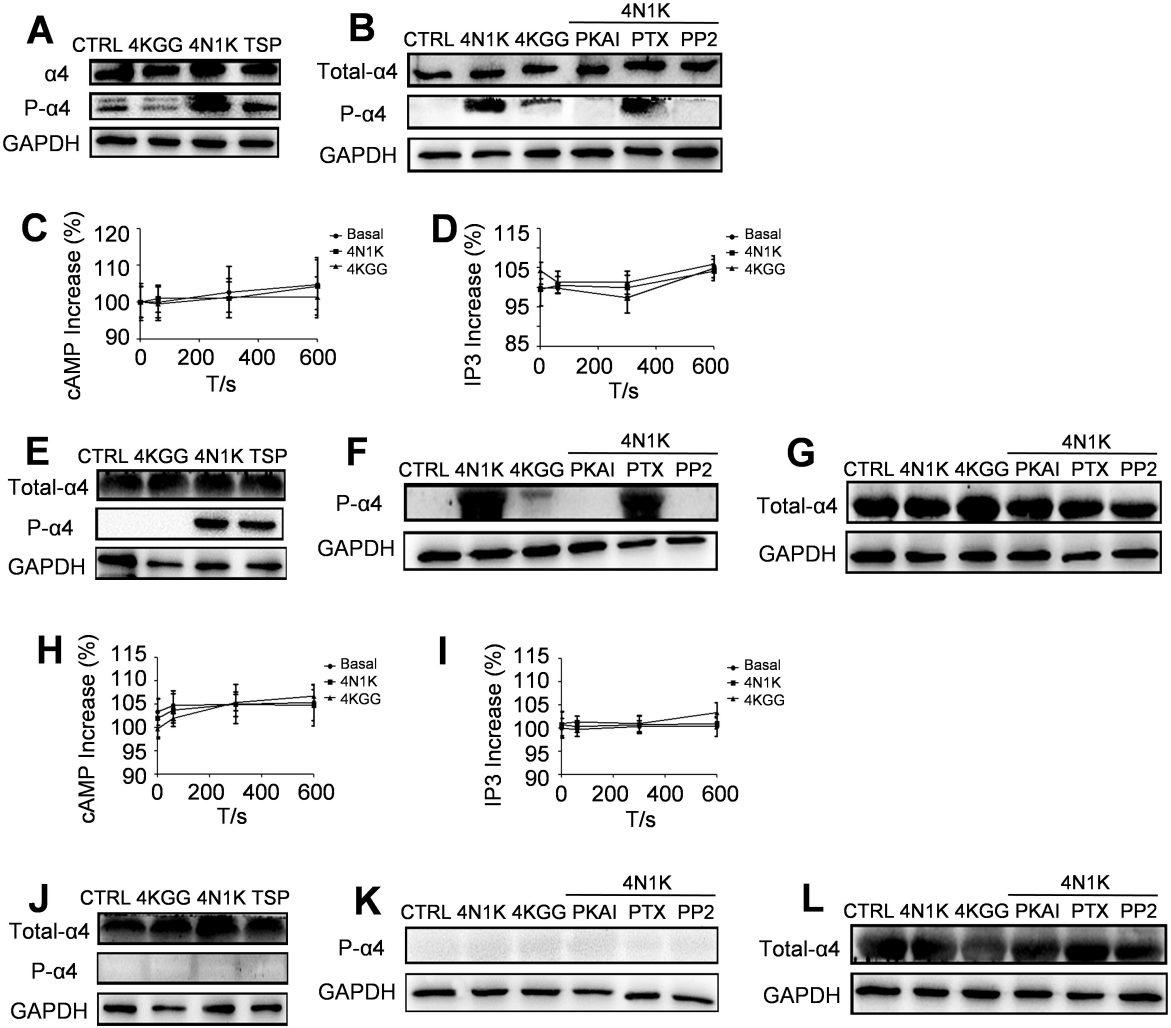
Inside-out signaling pathways lead to integrin α4β1 activation. A monoclonal antibody α-PSα4 was used for the detection of the Ser988-phosphorylated form of the α4 cytoplasmic domain. **(A)** Peptide 4N1K and TSP increased the amount of phosphorylated α4 subunit in Jurkat cells whereas peptide 4KGG had no effect. **(B)** PKAI and PP2 inhibited 4N1K effect on α4 subunit phosphorylation in Jurkat cells whereas PTX had no effect. Peptide 4N1K and 4KGG treatment did not change the amount of intracellular cAMP **(C)** or IP3 **(D)** in human Jurkat cells (n=3, three replicate samples for each experimental condition). Results are presented as the mean ± SD. For CD3+ T cells isolated from wild-type rats, peptide 4N1K and TSP increased the amount of phosphorylated α4 subunit whereas peptide 4KGG had no effect **(E)**. PKAI and PP2 inhibited 4N1K effect on α4 subunit phosphorylation whereas PTX had no effect **(F)**. The amount of intracellular cAMP **(H)** or IP3 **(I)** was not significantly changed in wild-type rat CD3+ T cells under treatment with peptide 4N1K, 4KGG, or without peptide treatment (n=3, three replicate samples for each experimental condition). Results are presented as the mean ± SD. For CD3+ T cells isolated from *Cd47* knockout rats, the amount of phosphorylated α4 subunit was undetectable whether the cells were treated with peptide 4N1K, TSP **(J)**, or 4N1K in combination with PKAI, PTX, or PP2 **(K)**. The total amount of integrin α4β1 was not changed under peptide 4N1K or its combination with PKAI, PTX, or PP2 treatment for CD3+T cells isolated from wild-type rats **(G)** and *Cd47* knockout rats **(L)**.

Phosphorylation of the integrin α4 subunit cytoplasmic domain modulated its interaction with the cytoskeletal protein paxillin (22), and paxillin is an important cytoskeletal protein for cell adhesion and mobility induced by integrin α4β1 (23). Although the phosphorylation of the α4 subunit cytoplasmic domain at the basal level was undetectable by western blot, 4N1K or thrombospondin (TSP) treatment of Jurkat cells induced the cytoplasmic domain phosphorylation of the α4 subunit (**Figure 6A**).

Previous research indicated that PKA played a central role in the cytoplasmic domain phosphorylation of the integrin α4 subunit. In addition, the cytoplasmic domain phosphorylation of integrin α4 subunits was shown to be Gi protein-dependent, and the Src kinase in the downstream signaling pathway of Gi protein was necessary for this process (14). An examination in this study revealed that the cytoplasmic domain phosphorylation of integrin α4 subunit was completely blocked by the PKA inhibitor PKAI (**Figure 6B**). In contrast, the cytoplasmic domain phosphorylation of the α4 subunit was not affected by the Gi protein inhibitor pertussis toxin PTX (**Figure 6B**). The CD47-induced signaling pathways did not cause changes in the overall levels of cAMP and IP3, which are the second messengers for the two principal signal transduction pathways involving the G protein-linked receptors: the cAMP signal pathway and the phosphatidylinositol signal pathway (24) (**Figures 6C, D**). Furthermore, the Src kinase family inhibitor PP2 markedly repressed the cytoplasmic domain phosphorylation of the α4 subunit induced by 4N1K in Jurkat cells (**Figure 6B**).

4N1K or TSP treatment of wild-type rat CD3+ T cells noticeably increased the phosphorylation of the α4 subunit cytoplasmic domain (**Figure 6E**). Co-incubation with PKAI or PP2 substantially reduced the phosphorylation of the cytoplasmic domain of the α4 subunit induced by 4N1K, while there was no effect with PTX co-incubation (**Figure 6F**). Correspondingly, cAMP and IP levels in wild-type rat T cells did not vary under treatment with 4N1K, 4KGG, or without peptide treatment (**Figures 6H, I**). In *Cd47*-deficient rat CD3+ T cells, intracellular phosphorylation of the α4 subunit cytoplasmic domain was not detected under all the above conditions (**Figures 6J, K**). The α4 subunit expression levels within T cells of wild-type rats (**Figure 6G**) and *Cd47* knockout rats (**Figure 6L**) did not change under 4N1K or 4N1K together with PKAI, PTX or PP2 treatments.

Peptide 4N1K treatment prominently increased the wild-type rat CD3+ T cell adhesion and migration towards the immobilized VCAM-1. 4N1K-stimulated cellular adhesion and migration were suppressed by co-incubation with PKAI or PP2, while co-incubation with PTX did not show an inhibitory effect (**Supplementary Figure S7**).

These results indicate that the activation of integrin α4β1 by CD47 or the phosphorylation of the α4 subunit cytoplasmic domain in T cells were Gi protein-independent, and PKA and Src kinase were the key enzymes in these activation processes.

### 2.7 CD47 antibody, PKA inhibitor, and Src inhibitor alleviate RA symptoms

**Figure 7:**
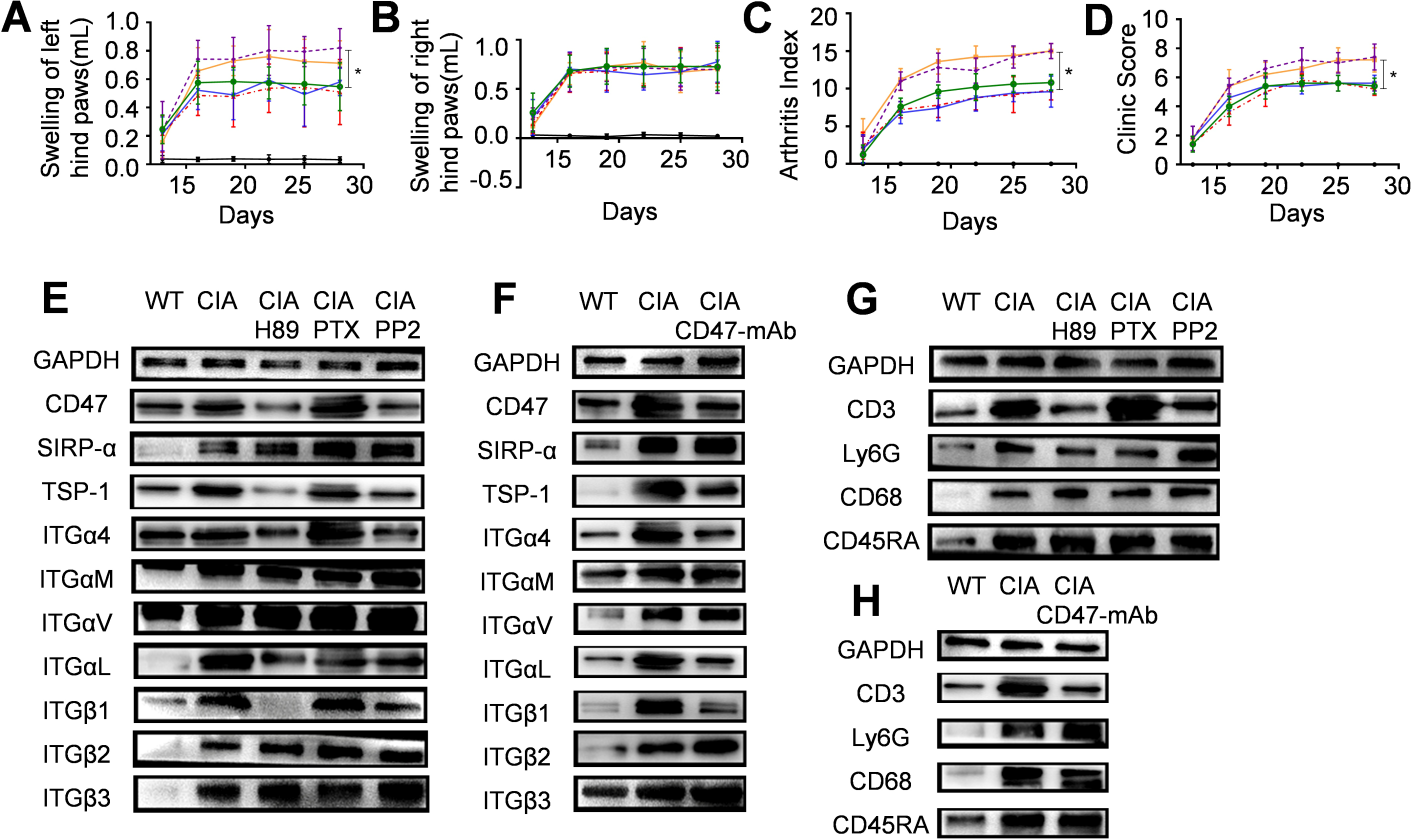
The therapeutic effect of local injection of CD47 antibody, PKA inhibitor, G protein inhibitor, or Src kinase inhibitor on arthritis development. Six groups of wild-type rats were included (n=5) and for panels **(A–D)** the solid black line stands for the normal rat group, the solid orange line for the model group, the dashed red line for the CD47 antibody treatment group, the solid blue line for the PKA inhibitor (H89) treatment group, the dashed purple line for the G protein inhibitor treatment group and the green line for the Src kinase inhibitor (PP2) treatment group. **(A–D)**. Swelling of hind left paws **(A)** and right paws **(B)**, arthritis score **(C)** and clinic score **(D)** of rats during the experiment were shown. On day 28 of the animal experiment, rats were sacrificed and synovial tissues of the hind left paws were taken and used as samples for western-blot analysis. Photos of western-blot for CD47, SIRP-α, TSP-1, integrin subunit α4, αM, αv, αL, β1, β2, and β3 under PKA inhibitor H89, G protein inhibitor PTX, and Src kinase inhibitor PP2 treatment **(E)** and CD47 antibody treatment **(F)** were shown. Photos of western-blot for markers of T cells (CD3), neutrophils (Ly6G), macrophages (CD68), and B cells (CD45RA) in synovial membranes of rats under PKA inhibitor H89, G protein inhibitor PTX, and Src kinase inhibitor PP2 treatment **(G)** and CD47 antibody treatment **(H)** were shown.

Next, we locally injected into CIA rats the CD47 antibody or inhibitors of key pathways in which CD47 activated integrin α4β1 (phosphorylating the α4 subunit cytoplastic domain) in T cells, and examined their effects on arthritis development. Experimental data showed that subcutaneous injection at the bottom of the left hind paw with the CD47 antibody, PKA inhibitor H89, or Src kinase inhibitor PP2 resulted in a significantly lower degree of swelling of the left hind paw compared with the WT-CIA group (subcutaneous injection of PBS after modeling), whereas subcutaneous injection of a G protein inhibitor (PTX) did not induce this effect (**Figure 7A**). The arthritis score (**Figure 7C**) and clinical score (**Figure 7D**) also showed similar outcomes. The animal groups did not differ in the swelling of the right hind paw as a control (**Figure 7B**). Western blot analysis of the left hind paw joint synovial tissues revealed that following treatment with the PKA inhibitor H89 or Src kinase inhibitor PP2, the expression of CD47, TSP-1, and integrin subunits α4, αL, and β1 noticeably decreased compared with the WT-CIA group, while the expression of SIRP-α and integrin subunits αM, αv, β2, and β3 showed minimal change (**Figure 7E**). Local injection of CD47 antibody also had similar results (**Figure 7F**). Furthermore, after treating with the PKA inhibitor H89, Src kinase inhibitor PP2, or CD47 antibody, the expression of the T cell marker molecule CD3 decreased in the left hind paw joint synovial tissue of the experimental animals, while the expression of the neutrophil marker (Ly6G), macrophage marker (CD68), and B cell marker (CD45RA) did not change significantly (**Figures 7G, H**). These results paralleled the previous findings showing that *Cd47* deficiency remarkably reduced the T cell infiltration into joint synovial tissues, and that the *Cd47* knockout significantly decreased the levels of TSP-1, CD47, and integrin subunits α4, αL, and β1 in the synovial tissue of model animals. In western blot assays, the levels of various molecules in the left hind paw joint synovial tissues of the animals treated with G protein inhibitor PTX did not significantly differ from those of the WT-CIA group. These experimental results suggested the TSP-1-CD47 interaction and that the key enzymes (PKA and Src) in the intracellular signaling pathways activating integrin α4β1 may become potential targets for RA therapeutics.

## 3 Discussion

**Figure 8:**
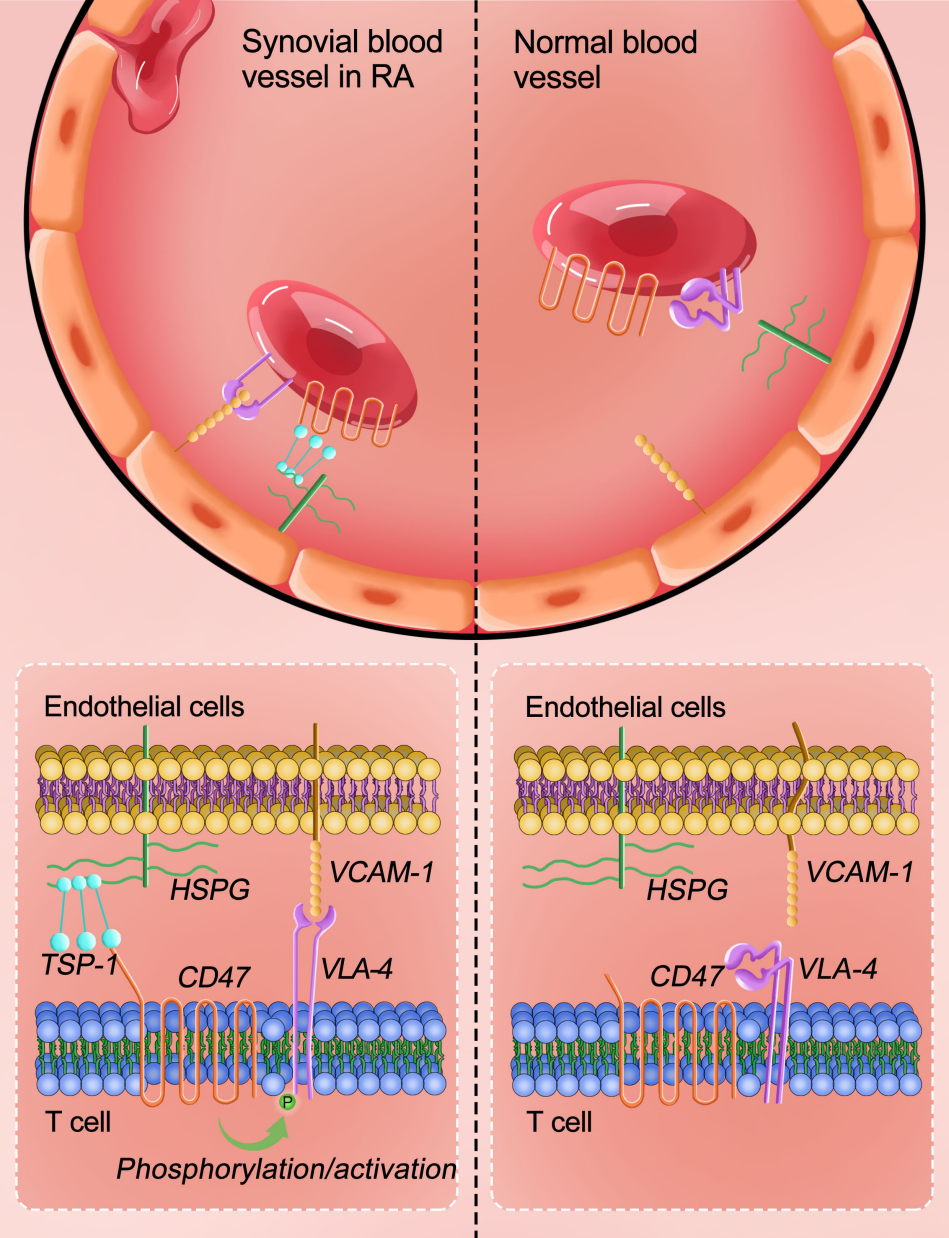
“TSP-1-CD47-integrin α4β1” trimolecular co-action model to explain the important role of CD47 in RA. CD47 expressed on the surface of T cells interacts with TSP-1 scattered on the blood vessel to promote integrin α4β1 (on T cell surface) interaction with VCAM-1 present on the surface of vascular endothelial cells, thereby promoting T cells infiltration into the joint synovial tissue and accelerating arthritis development.

During the occurrence and development of RA, angiogenesis occurs in the synovial tissue, and the expression of VCAM-1 and TSP-1 in the synovium increases, which is in line with abundant expression of TSP-1 in rheumatoid synovium (11). Experiments in this study showed that during CIA development, TSP-1 interacts with CD47 on the surface of T cells, and through intracellular signaling pathways such as PKA and Src kinase, it activates integrin α4β1 on the surface of T cells. The activated integrin α4β1 binds to VCAM-1 on the surface of endothelial cells with high affinity, increasing T cell infiltration into the synovial tissue and releasing cytokines such as TNF-α, IL-1, IL-6, and IL-17, exacerbating synovial inflammation. Based on these results, this study proposes a three-molecule model of “TSP-1, CD47, and integrin α4β1” to explain the important role of CD47 in collagen-induced arthritis in rats. Based on the results of GEO data analysis and with Jurkat cells, the three-molecule model may also explain the important role of CD47 in rheumatoid arthritis (**Figure 8**).

*Cd47* deficiency and re-expression Jurkat cells were used to investigate the contribution of CD47 to T cell arrest on inflammatory vascular endothelium (15). Memory or effector T cells express increased levels of adhesion molecules compared to naïve T cells (25), and they are the main T cells that adhere to and migrate through endothelium of inflamed areas (26). Jurkat cells express large amount of integrin α4β1 and can be compared to the memory sub-population of mature T cells (27). T cell express high levels of CD47 and no difference in CD47 expression can be found between T cell populations, whether resting or activated T cells and whether naïve or memory T cells (15). Therefore, this study used Jurkat cells as a cell model to study TSP-1-CD47-integrin α4β1 co-action in the occurrence and development of RA.

TSP family members can mediate cell binding to CD47 via their shared C-terminal domain (28, 29). Peptide 4N1K, with a sequence KPFYVVMWKK, is derived from the C-terminal globular domain that was the main site responsible for TSP-mediated cell adhesion (30, 31). 4N1K has been shown to regulate integrin-mediated adhesive functions and some studies showed that 4N1K could decrease cell adhesion to immobilized VCAM-1 (15), laminin (30), collagen (32) and fibronectin (33). However, other studies showed that 4N1K could increase adhesion to TSP (34) and immobilized VCAM-1 (14). For example, a previous study found that when 4N1K is co-plated with VCAM-1 on the bottom of a flat surface, it increases T cell adhesion to the surface, while free 4N1K reduces T cell adhesion (15). In this study, experiments confirmed that free 4N1K enhances T cell adhesion and migration, and it also increases the amount of active integrin α4β1 on the surface of T cells (**Figures 5E, F, K**). This activation is achieved by phosphorylation of the intracellular domain of integrin α4 (**Figure 6A**). The discrepancy between the results of these two studies may be due to the use of different cell types and culture conditions. The previous study used wt-CD47POS cells, Jurkat cells with *Cd47* deficiency that were transfected with intact CD47 molecules. In contrast, we used wild-type Jurkat cells (cultured in RPMI-1640 medium containing 10% fetal bovine serum). Under this culture condition, the prerequisite for CD47 aggregation or cross-linking to induce integrin α4β1 activation may be naturally satisfied. Therefore, in our study, free 4N1K activated integrin α4β1 by interacting with CD47. Furthermore, studies using *Cd47*-deficient cell lines found that 4N1K could mediate effect that is independent of CD47 expression, including 4N1K-mediated platelet aggregation (35) and 4N1K-induced cell adhesion (36, 37). In our study CD3+ T cells form both wild-type and *Cd47* knockout rats were used to investigate 4N1K-mediated effect. It was found that 4N1K can significantly increase wild-type rat T cell adhesion (**Supplementary Figure S6A**) and migration (**Supplementary Figure S6C**), significantly increase percent of extended form integrin α4β1 on T cell surface (**Supplementary Figure S6I**), significantly increase the amount of phosphorylated form integrin α4 subunit (**Figure 6E**) whereas 4N1K had no such effect on *Cd47* deficient T cells (**Supplementary Figures S6B, D, M, O**, **Figure 6J**). These results confirmed that the 4N1K-mediated effect in this study was CD47-dependent.

In sickle erythrocytes, CD47 interacts with extracellular ligands to activate integrin α4β1 in a Gi protein-dependent manner. This is because the Gi protein inhibitor pertussis toxin blocks intracellular phosphorylation of the α4 subunit induced by CD47 (14). In this study, activation of integrin α4β1 by CD47 is found to be Gi protein-independent. The Gi protein inhibitor pertussis toxin did not affect intracellular phosphorylation of the α4 subunit after interaction between 4N1K and CD47 in human and mouse T cells (**Figures 6B, F**), nor did it inhibit adhesion or migration of wild-type mouse T cells to immobilized VCAM-1 after interaction with 4N1K (**Supplementary Figures S7B, D**). Animal experiments also demonstrated that local injection of pertussis toxin did not alleviate RA symptoms (**Figure 7**). These experiments suggest that the intracellular signaling pathway activated by TSP-1 interacting with CD47 to activate integrin α4β1 may be different in different cell types.

Scientific research has shown that both integrin α4β1 and integrin αLβ2 on the surface of T cells play important roles in the process of T cells entering inflammatory sites in the circulatory system (21, 38, 39). Our study found that after TSP-1 (4N1K), a ligand of CD47, acts on CD47 on the surface of T cells in blood vessels, CD47 only forms a complex with integrin α4β1, activates integrin α4β1, and enhances T cell adhesion and migration to VCAM-1 (**Figure 5**). This process does not involve integrin αLβ2 (**Supplementary Figure S5**).

This study observed that after interacting with TSP-1, CD47 on T cells activates integrin α4β1 through intracellular signaling pathways such as PKA and Src kinase. It was further observed that subcutaneous injection of CD47 antibodies or inhibitors of PKA and Src kinases into the left hind paw of wild-type rats effectively alleviated arthritis symptoms. This not only demonstrates the establishment of the proposed three-molecule model of “TSP-1, CD47, and integrin α4β1” in collagen-induced arthritis at the whole animal level, but also suggests that the interaction among these three molecules plays an important role in collagen-induced arthritis. Blocking the TSP-1-CD47 interaction or inhibiting the activation of integrin α4β1 on T cells by CD47 can serve as important therapeutic strategies for collagen-induced arthritis. This suggests that monoclonal antibodies against CD47 (if their side effects can be resolved), as well as inhibitors of PKA kinases and Src kinases, particularly specific inhibitors of Src kinase, may reduce T cell infiltration into inflammatory sites and thereby treat rheumatoid arthritis. Based on information from the Cortellis platform Drug Discovery Intelligence, there are currently no CD47 antibodies, PKA inhibitors, or Src inhibitors used to treat rheumatoid arthritis. The results of this study provide new ideas for the development of potential therapeutic targets and drugs for RA. While the manuscript provides evidence for the role of CD47 in RA pathogenesis, further studies are needed to address the potential immune suppression risks and off-target effects of CD47 blockade. Future research should focus on developing more selective CD47 inhibitors and exploring combination therapies to maximize therapeutic benefits while minimizing adverse effects.

Worth of mention here is that gene editing technology was employed in this study to delete a 31-bp segment in the second exon of the *Cd47* gene with the aim of generating *Cd47* knockout rats. Sequencing confirmed the successful deletion of the 31-bp segment at the genomic DNA level, indicating successful gene disruption. However, Western blot analysis in **Supplementary Figure S3C** revealed bands in the *Cd47* gene-edited rat samples that corresponded to a molecular weight similar to that of CD47 protein (comprising 303 amino acid residues). Additionally, mass spectrometry analysis of protein expression profiles in the tail tissue of the *Cd47* gene-edited rats detected three characteristic CD47 peptide fragments (corresponding to amino acid residues 62-71, 85-92, and 289-295). Furthermore, RNAseq analysis of mRNA expression profiles in the synovial tissue of the *Cd47* gene-edited rats revealed the presence of *Cd47* mRNA (**Figure 3**), which was also detected in RT-PCR experiments (**Figure 3**). However, in the *in vivo* and *in vitro* functional studies of this project, including animal modeling experiments (**Figure 2**), flow cytometry analysis (**Supplementary Figure S6**), cell adhesion and migration assays (**Supplementary Figure S6**), and Western blot analysis (**Figure 6**), all confirmed the disruption of CD47 protein function. Theoretically, the deletion of the 31-bp segment in the second exon of *Cd47* would result in a frameshift mutation, leading to incorrect translation downstream with disrupted protein function. The mRNA product of the frameshift mutation would undergo nonsense-mediated mRNA decay (NMD) in mice, although not reaching 100% degradation according to the literature (40). Hence, the truncated mRNA product would still be detectable by RT-PCR and RNAseq, albeit with significantly reduced expression levels. Residual truncated mRNA may undergo unknown expression regulation mechanisms, leading to the translation of truncated peptide fragments, which could explain the faint, smaller-sized band detected by Western blot (as the antibody can detect at least the peptide region upstream of the 31 bp deletion). The identification of partial peptide fragments by mass spectrometry suggests their translation from the truncated mRNA. Functional assays demonstrated that these detected residual peptide segments lack CD47 protein activity, confirming the loss of CD47 function. Based on these considerations, this study describes the gene-edited rats as *Cd47* knockouts or having CD47 functional deficiency.

## 4 Conclusion

This study proposes a three-molecule model of “TSP-1, CD47 and integrin α4β1” to confirm that CD47 plays an important role in the occurrence and progression of collagen-induced arthritis, a typical animal model of rheumatoid arthritis. Blocking the TSP-1-CD47 interaction or inhibiting CD47-activated integrin α4β1 on T cells could be a potential therapeutic strategy for rheumatoid arthritis.

## 5 Materials and methods

### 5.1 Bioinformatic analysis

Expression profiles of CD47, TSP1, SIRP-α, and integrin subunits (α4, αM, αv, αL, β1, β2, and β3) in the synovial tissue or peripheral blood samples of RA patients and healthy people were analyzed and established based on published NCBI-GEO profiles (GPL96, GPL570, GPL11154, GPL1708, GPL10558, GPL91, GPL6947, GPL570, GPL20171, and GPL13158 platforms; for synovial tissue sample RA=230, Normal=59; for peripheral blood sample RA=1238, Normal=120).

Gene chip data from human synovial tissue and peripheral blood tissue in public databases were retrieved and analyzed. All the GEO project data related to “Homo sapiens” and “synovial or Blood” from the NCBI database were retrieved and the corresponding CEL files for each GEO dataset were downloaded. These CEL files were then subjected to preprocessing using the affy package in R language. Subsequently, the integrated data were subjected to batch correction using the sva package in R language. Differential expression analysis between the RA group and the normal group was conducted utilizing the limma package. Only dual-channel chip data were retained for further analysis.

Violin Plot, Heatmaps, and tabulated values (LogFC and adjusted P values) were calculated and presented. Threshold values for differential expression are logFC≥0.263 and adj.P.Val ≤ 0.01.

The linearity of CD47 and relevant integrin subunit α4, αM, αL, β1, β2, SIRP-α, and ICAM-1 was analyzed based on the published GEO profiles (NCBI/GEO/GSE 55457/77298/55235).

### 5.2 Cell culture and reagents

Human Jurkat cells (SCSP-513) were purchased from the Cell Bank of the Chinese Academy of Sciences (Shanghai, China) and cultured in RPMI-1640 medium (Gibco, Grand Island, NY) with 10% FBS (Life Technologies, Carlsbad, CA) and 1% penicillin/streptomycin (Invitrogen, USA). The non-tumorigenic rat CD3+ T cells were isolated from wild-type or *CD47* knockout rats using anti-PE MicroBeads and PE-Anti-CD3 antibodies (cat log: 130-048-801 and 130-103-130, Miltenyi, China). Cells were cultured in RPMI-1640 culture medium with 10% FBS and 1% penicillin/streptomycin containing recombinant rat IL-2 (400-02, Peprotech).

Complete Freund’s adjuvant (M. tuberculosis H37RA, 10 mg·mL^−1^) and bovine type II collagen were purchased from Chondrex (Redmond, WA, USA). The kits for detection of rat TNF-α (ml002859), rat IL-6 (ml102828), rat rheumatoid factor (ml059541), anti-CCP antibody (ml059150), anti-collagen antibody (ml058275), human cAMP (ml064278), human IP3 (ml060362), rat cAMP (ml002907) and rat IP3 (ml059408) were from Shanghai Meilian Biotechnology Co., Ltd. (Shanghai, China). TRIzol was purchased from Invitrogen Trading Co., Ltd (Waltham, MA, USA). A PrimeScriptTM RT reagent kit with gDNA Eraser and SYBR^®^ Premix Ex Taq™ II (Tli RNaseH Plus) was purchased from TaKaRa (Kyoto, Japan). HRP conjugated anti-GAPDH mouse mAb (70-ab011-040), HRP conjugated goat anti-rabbit IgG (70-GAR007), and HRP conjugated goat anti-mouse IgG (70-GAM007) were purchased from Multi sciences (Hangzhou, China). Monoclonal antibodies specific for CD47 (ab108415), SIRP-α (ab191419), and polyclonal antibodies specific for integrin subunit αM (ab133357) were purchased from Abcam (Cambridge, UK). A monoclonal antibody specific for integrin subunit β1 (34971S) was from Cell Signaling Technology (Danvers, MA, USA). Monoclonal antibodies specific for integrin subunit α4 (sc-376334), β2 (sc-8420), p-α4 (sc-23943), and IL-23 (sc-271279) were from Santa Cruz Biotechnology (Santa Cruz, CA, USA). Polyclonal antibodies specific for IL-17A (DF6127), IL-1β (AF5103), IL-12A (AF5133), and IL-6 (DF6087) were from Affinity. Polyclonal antibodies specific for FoxP3 (22228-1-AP) and TGF-β1 (21898-1-AP) were from ProteinTech (Wuhan China). PKAI (476485) and PTX (516560) were from Merck&Millipore (Burlington, MA, USA). PP2 (13198) was from Cayman (Ann Arbor, MI, USA). Human Thrombospondin-1 (SRP4805) was from Sigma-Aldrich (St. Louis, MO, USA).

Peptide 4N1K (KRFYVVMWKK) and 4KGG (KRFYGGMWKK) were synthesized by GL Biochem Co., Ltd. (Shanghai, China) with a purity of more than 95%.

### 5.3 Establishment of collagen-induced arthritis in rats and detection

#### 5.3.1 Animals and experiment design

Wild-type female Sprague Dawley (SD) rats (6 to 8 weeks, 180-200 g) were purchased from the sippr-BK Experimental Animal Center (Shanghai, China) and were used for induction of the CIA model. Animals were cared for according to the Provisions and General Recommendation of Chinese Experimental Animals Administration Legislation. All experiments had been performed in compliance with the Guide for the Care and Use of Laboratory Animals of China Pharmaceutical University.

Before CIA induction, bovine type II collagen was dissolved in 0.1 M acetic acid solution at 2 mg/mL and was kept at 4°C overnight. The next day, the collagen solution was mixed with the same volume of Complete Freund’s adjuvant (CFA) and the mixture was completely emulsified. 200 μL emulsion containing 200 μg collagen was slowly and subcutaneously injected into the rat tail which was 1.5 cm from the root of the tail. On day 21, a second injection was performed. For CIA induction in wild-type rats (**Supplementary Figure 2**), there were two groups of rats including the normal wild-type and the model wild-type rats (n=6). For CIA induction in *Cd47* knockout rats (**Figure 2**), there were four groups of rats including the normal wild-type group, the normal *Cd47* knockout group, the model wild-type group, and the model *Cd47* knockout group (n=5). For CIA induction in wild-type rats to evaluate the therapeutic effect of a CD47 antibody, a PKA inhibitor, a G protein inhibitor, and an Src kinase inhibitor on RA syndrome development (**Figure 7**), there were six groups including the normal group, the model group, the CD47 antibody treatment group, the PKA inhibitor H89 treatment group, the G protein inhibitor PTX treatment group, and the Src kinase inhibitor PP2 treatment group (n=5). From the 1^st^ day of CIA induction rats in the corresponding group were subcutaneously injected in the left hind paw with 0.05 ml solution which contained 200 μg CD47 antibody (MIAP410), 260 ng PKA inhibitor H89, 0.5 μg G protein inhibitor PTX or 120 μg Src kinase inhibitor PP2. CD47 antibody was administered every other day and the other reagents were administered every day until day 28^th^. All experiments had been performed in compliance with the Guide for the Care and Use of Laboratory Animals. After the animals had been sacrificed under sodium pentobarbital anesthesia, left and right hind paw tissues were collected.

For assessment of Arthritis Symptom, from day 13, the swelling of the left and right hind paws was measured once every 3 days. The swelling of paws was determined by water volume (the displacement method): swelling (mL) = paw thickness (mL) tested value - paw thickness (mL) original value. The arthritic index was assessed once every 3 days from the 13^th^ day. Arthritis severity was scored by grading each paw from 0 to 4 based on erythema, swelling, and deformity of the joint: 0 = no erythema or swelling; 1 = slight erythema or swelling of one of the toes or fingers; 2 = erythema and swelling of more than one toe or finger; 3 = erythema and swelling of the ankle or wrist; 4 = complete erythema and swelling of toes or fingers and ankle or wrist, and the inability to bend the ankle or wrist. All four paws were scored and the maximum possible score per rat was 16. The clinical score assessment for the systematic inflammation lies between 0 and 8 for each animal (sum of 1 = nose, 1 = left ear, 1 = right ear, 1 = left paw, 1 = right paw, 1 = left hind paw, 1 = right hind paw, 1 = tail). The body weight of the rat was measured every 3 days with a precision of 0.1 g.

For detection of RF, anti-CCP antibodies, anti-collagen II antibodies, and TNF-α in rat circulation, rat blood was taken from the vein of the eyes on day 28. Blood cells were removed by centrifugation and the serum was used for detection with commercially available kits. For detection of leukocytes, neutrophils, and platelets in rat circulation, rat blood was analyzed with a blood cell analyzer (mindray, CAL6000).

#### 5.3.2 Western-blot assay of protein expressions in synovial tissues

Western-blot assay was performed to verify the expression of the key protein CD47, SIRP-α, TSP-1, integrin subunit α4, αM, αv, αL, β1, β2, β3, IL-1β, IL-12A, TNF-α, IL-6, IL-17A, IL-23, FoxP3, and TGF-β. GAPDH was used as a quantity control. After the sacrifice of the experimental rats, synovial tissue was surgically removed. The synovial tissues were put in a lysate reagent (Wanlei Biotech Co., Ltd.) and were homogenized. The homogenate was centrifuged at 14,000 g for ten minutes and the supernatant was collected. These steps were performed at 4°C and the lysate was pre-cooled at 4°C. Protein content was quantified with a BCA method. Proteins in the synovial sample were separated on a 12% SDS-polyacrylamide gel (SDS-PAGE), and the protein bands were transferred to a 0.22 µm aperture polyvinylidene fluoride (PVDF) membrane. Then the PVDF membrane was incubated with a first antibody (including anti-CD47, SIRP-α, TSP-1, integrin subunit α4, αM, αv, αL, β1, β2, β3, IL-1β, IL-12A, TNF-α, IL-6, IL-17A, IL-23, FoxP3, TGF-β or GAPDH antibodies) that has been properly diluted according to the instructions of the products. Next, the membrane was incubated with a 1:2000 dilution of secondary antibodies (HRP conjugated goat anti-rabbit IgG or HRP conjugated goat anti-mouse IgG) and the bands were visualized with an electro-chemiluminescence (ECL) reagent.

#### 5.3.3 RNA isolation and quantitative real-time PCR analysis

Total RNA was extracted using TRIzol following the manufacturer’s specifications. cDNA was synthesized from the total RNA using PrimeScript™ Kit. Transcription levels were measured in duplicate by PowerUp™ SYBR™ Green Master Mix. Nuclear/cytoplasmic fractionation was performed using the PARIS Kit (Ambion) following the manufacturer’s instructions. Relative expression levels of lncRNA and mRNA were normalized to GAPDH expression. The primer sequences for CD47 (5’-gctcacactcatcgtggttg-3’, 5’-caaggacatagcccagcact-3’), ACTB (5’-cgcgagtacaaccttcttgc-3’, 5’-ccttctgacccatacccacc-3’), SIRP-α (5’-ggacattcattctcgggtcat-3’, 5’-ttccattctccagccagttc-3’), TSP-1 (5’-gtgaccctggacttgctgtag-3’, 5’-agtatccctgagcccttgtg-3’), integrin subunit α4 (5’-caaagaggtcccaggctaca-3’, 5’-gatgcccaaggtggtatgtg-3’), integrin subunit αM (5’-ggagtgtgtttgcgtgtcaa-3’, 5’-tgccgttctttgtttcatca-3’), integrin subunit αv (5’-cgttagggcaggtaggattg-3’, 5’-aaagtcccgctgtgtaaatga-3’), integrin subunit αL (5’-aggtgacgggactcaacaac-3’, 5’-acttggcttcagggcatttc-3’), integrin subunit β1 (5’-atcccagcaagtcccaagt-3’, 5’-ccacagacacactctccattgt-3’), integrin subunit β2 (5’-tcaagaagttgggtggtgatt-3’, 5’-ctggttggagttgtcggttag-3’) and integrin subunit β3 (5’-gggctgtcctgtatgtggtag-3’, 5’-atttggctctggctcgttct-3’) were used.

#### 5.3.4 H&E staining and immunofluorescence analysis

ServiceBio. (Wuhan, China) were commissioned to perform H&E staining, immunostaining and immunofluorescence analysis.

The left and right hind paws were collected and decalcified by 5.0% ethylene diamine tetraacetic acid (EDTA) in 10% formaldehyde at room temperature. The liquid was changed twice a week for eight weeks. The tissues were dehydrated and embedded in paraffin, and serial sections (4 µm) were cut and mounted on glass slides. The sections were stained with hematoxylin-eosin (HE) for examination under a microscope (Olympus, Tokyo, Japan) with a magnification of 200 times.

The expression of CD34, VCAM-1, and ICAM-1 on rat ankle joint sections was analyzed with an immunostaining method. The sections were mounted on glass slides. After dewaxing and alcohol gradient hydration, the slides were pre-incubated in 3% BSA to reduce nonspecific staining at room temperature. The slides were incubated with specific rabbit anti-rat polyclonal antibodies against CD34 (GB11013), ICAM-1 (GB11106), or VCAM-1 (GB11336) overnight at 4te After washing with PBS, the slides were incubated with HRP conjugated goat anti-rabbit IgG (H+L) (GB23303). The sections were washed with PBS and stained with 3,3-diaminobenzidine (DAB) until brown color appears for the positive sample by observation under microscope. Then, they were stained with hematoxylin, dehydrated, and sealed with neutral resin for examination under a microscope (Nikon E100, Japan) with a magnification of 200 times. The images were analyzed using the ImageJ 1.51K software (NIH, USA). All the sections were scored with the same standard and the score was based on staining intensity and percentage of positive cells.

The expression of CD3 (a T cell marker), Ly6G6d (a neutrophil marker), CD68 (a macrophage marker), and CD45RA (a B cell marker) on rat ankle joint sections were analyzed with an immunofluorescence staining method. The sections were mounted on glass slides. After dewaxing and alcohol gradient hydration, the slides were pre-incubated in 3% BSA to reduce nonspecific staining at room temperature. The slides were incubated with a specific rabbit anti-rat CD3 monoclonal antibody (GB13014), rabbit anti-rat CD68 (GB11067), rabbit anti-rat CD45RA (GB11066), or rabbit anti-rat Ly6G6d (GB11229) polyclonal antibodies overnight at 4te After washing with PBS, the slides were incubated with Cy3 conjugated goat anti-rabbit IgG (H+L) for CD45RA staining and incubated with FITC conjugated goat anti-rabbit IgG (H+L) for CD3, CD68, or Ly6G6d staining for 50 min at 370 After washing with PBS, the sections were stained with DAPI for 10 min at room temperature in dark. Sections were dehydrated and sealed with neutral resin for observation under a fluorescence microscope (Nikon Eclipse C1) and images taken. Fluorescent images were analyzed using the ImageJ 1.51K software (NIH, USA). All the sections were scored with the same standard and the score was based on staining intensity and percentage of positive cells.

For simultaneous observation of the red signal for CD3 and CD4 and the green signal for IL-23R, CD25, CD4, and CD8, a similar procedure was used as described above. Briefly, sections were incubated with rabbit anti-rat CD4 (GB13064-2), rabbit anti-rat IL-2 receptor alpha (GB11612), or rabbit anti-rat IL-23R (GB11660) polyclonal antibodies, or a rabbit anti-rat CD8 (GB13429) monoclonal antibody overnight at 4te After washing with PBS, the slides were incubated with HRP-conjugated goat anti-rabbit IgG (H+L) for 50 min at 370 After washing with PBS, the sections were incubated with FITC-TSA (G1222) for 10 min at room temperature in dark. Then the bound first and secondary antibodies were removed by incubation of the sections in EDTA containing buffer (pH 8.0). Then the sections were stained with the first antibodies for a second antigen. For sections of IL-2 receptor alpha and IL-23R staining, the sections were incubated with rabbit anti-rat CD4 (GB13064-2) polyclonal antibodies overnight at 4t in dark. For sections of CD4 and CD8 staining, the sections were incubated with a rabbit anti-rat CD3 monoclonal antibody (GB13014) overnight at 4t in dark. After washing with PBS, the sections were incubated with Cy3 conjugated goat anti-rabbit IgG (H+L) (GB21303) for 50 min at room temperature in dark. After washing, the sections were stained with DAPI for 10 min at room temperature in dark. Fluorescent images were observed with a confocal laser scanning microscope (CLSM, LSM800, Zeiss, Germany) and analyzed using the ZEN imaging software. All the sections were scored with the same standard and the score was based on staining intensity and percentage of positive cells.

The antibodies used in immunostaining and immunofluorescence staining were all from ServiceBio. (Wuhan, China).

### 5.4 Generation of *Cd47* knockout rats

*Cd47* knockout rats were generated under SD rat gene background under SPF conditions by Nanjing Biomedical Research Institute of Nanjing University (NBRI). Project number: XM200765. Strain ID: T004140. Genetic background: SD. *Cd47* gene includes 9 exons. The second exon of *Cd47* was used as a sgRNA target and sgRNA primers were designed using the Cas9 target design software (http://crispr.mit.edu/). The sequence of sgRNA (5’→3’) was ACAATGGATACCCATG for S1 with PAM as AGG and TCTCAGACTTGCTCAA for S2 with PAM as AGG. After the design and clone of sg RNA vector, Cas9 mRNA and sgRNA were *in vitro* transcribed. 100 ng/μL Cas9 mRNA and 40 ng/μL sgRNA were micro-injected into fertilized eggs and the eggs were put back in the uterus of pseudo-pregnant rats. After DNA extraction from F0 rats and gene sequencing, positive F0 rats were selected and were crossed with wild-type rats of the same genetic background. Positive F1 rats were selected by DNA extraction and gene sequencing. Male and female positive F1 rats mated to generate F2 rats. Homozygous *Cd47* knockout F2 rats were selected by DNA sequencing and protein identification. Male and female homozygous *Cd47* knockout rats mated to generate more *Cd47* knockout rats for the experiments in the current study in comparison with wild-type rats of the same age. Sequence (5’→3’) for primers for gene sequencing were GTGTCTGTATTCCAGGTTCAGCTC for primer T1 and TCCAGATGGCCAGTCCACCT for primer T2.

During the breeding process, *Cd47* knockout rats did not exhibit any obvious differences from wild-type rats in terms of food intake, growth rate, body weight, and litter size.

### 5.5 Systemic protein expression in *Cd47* knockout rat and wild-type rat

CapitalBio Technology (Beijing, China) was commissioned to conduct this experiment according to the protocol. Briefly, rat tail tissues from a *Cd47* knockout rat and a wild-type rat were ground in liquid nitrogen and lysed using protein extraction buffer (8 M urea, 0.1% SDS) containing 1 mM PMSF and a cocktail of protease inhibitors (Roche, USA). BCA assay was used for protein concentration measurement. Relative quantification of proteins was performed by Tandem mass tag TMT6/10 (Pierce, USA) labeling, which uses different reporter ions (126-131 Da) as isobaric tags. After lyophilization, protein samples were dissolved in solvent A (2% ACN, pH10) and RPLC was developed on Xbridge PST C18 Column (130 Å, 5 μm, 250×4.6 mm) (Waters, USA) with a gradient of 5 to 95% solvent B (90% ACN, pH10). Fractions were collected and vacuum dried, and LC-MS/MS analysis was performed using a Q Exactive mass spectrometer (Thermo Scientific, USA). The peptide mixture was loaded onto an Acclaim C18 PepMap100 nano-Trap column (75 μm×2 cm, 2 μm particle size) connected to an Acclaim PepMap RSLC C18 analytical column (75 μm×25 cm, 2 μm particle size) (Thermo Scientific, USA). The nano-LC was coupled online with the Q Exactive mass spectrometer. Mass spectrometry analysis was performed with full scans (350-1,600 m/z) acquired at a mass resolution of 70,000 at 400 m/z in Q Exactive. The twenty most intense precursor ions were selected for MS/MS and detected at a mass resolution of 35,000 at m/z of 400 in the Orbitrap analyzer. Database searching against the NCBI’s RefSeq human protein sequence database was performed using proteome discoverer software (version 1.4) (Thermo Scientific, USA). Trypsin was used as the digesting enzyme with a tolerance of 2 missed cleavages. Fixed modifications include cysteine carbamidomethylation and TMT modifications (N-terminus and lysine residues) and variable modifications include methionine oxidation. For protein quantitation, unique peptides were used to quantify proteins.

### 5.6 RNAseq analysis of protein expression in the synovial membrane of *Cd47* knockout rat and wild-type rat

RNAseq analysis was performed by CapitalBio Technology (Beijing, China). Briefly, synovial membrane samples were prepared from the hind paws of rats in the four groups (n=3 for each group). For mRNA library construction and deep sequencing, RNA samples were prepared by using the TruSeq RNA Sample Preparation Kit according to the manufacturer’s protocol. Briefly, the poly-A containing mRNA molecules were purified from the 3 μg of total RNA by using poly-T oligo-attached magnetic beads. The cleaved RNA fragments were reversely transcribed into first-strand cDNA using random hexamers, followed by second-strand cDNA synthesis using DNA Polymerase I and RNase H. The cDNA fragments were purified, end-blunted, ‘A’ tailed, and adaptor-ligated. PCR was used to selectively enrich those DNA fragments that have adapter molecules on both ends and to amplify the amount of DNA in the library. The number of PCR cycles was minimized to avoid skewing the representation of the library. The library was qualified by Agilent 2100 bioanalyzer and quantified by Qubit and qPCR. The produced libraries were sequenced on the Novaseq 6000 instrument (Illumina).

### 5.7 Mechanism for CD47 engagement to activate integrin α4β1

#### 5.7.1 Immunoprecipitation and immunofluorescence detection

Human Jurkat cells were incubated in RIPA solution (Beyotime, P0013) which contains PMSF (Beyotime, ST506) on ice. After centrifugation, protein content in the supernatant was quantified and the supernatant was adjusted with PMSF containing RIPA solution. Cell lysate was incubated with an anti-CD47 antibody (SANTA CRUZ, SC-12730), an anti-integrin subunit α4 antibody (Novus, NBP2-50445) or an anti-integrin subunit β1 antibody (Abcom, ab24693) that have been properly diluted according to the instructions of the products. The solution was rotated at 4°C overnight. Then Protein G sepharose beads (Cell Signaling Technology, #37478) were added and after rotation of the tube at 4°C for 3 h, the beads were collected by centrifugation. After washing the beads with blank PMSF containing RIPA solution, loading buffer for SDS-PAGE was added to the beads and after incubation of the tube in boiling water for 5 min, the supernatant was collected by centrifugation. Routine western-blot analysis of the samples was performed with an anti-GAPDH antibody (Lianke Biotech, 70-Mab5465-040), an anti-CD47 antibody (SANTA CRUZ, SC-12730), an anti-integrin subunit α4 antibody (Novus, NBP2-50445), and an anti-integrin subunit β1 antibody (Abcam, ab24693) as first antibodies and with an HRP conjugated goat anti-mouse antibody (Abcam, ab205719) as a secondary antibody.

For laser confocal microscopy observation of CD47 and integrin subunit α4 signals, suspending human Jurkat cells were incubated with APC conjugated anti-CD47 antibodies (Biolegend, 323123), FITC conjugated anti-integrin subunit α4 antibodies (Biolegend, 304315), and Hoechst33342 (WLA042a, Wanlei Bio.) at 4°C for 1 h in dark. Then the cells were washed with pre-cooling PBS twice. Cells were put in a dish specific for a laser confocal microscope (150260, Thermo Scientific) and observed under a laser confocal microscope (LSM800, Zeiss).

For laser confocal microscopy observation of CD47 and integrin subunit αL signals, suspending human Jurkat cells were incubated with APC conjugated anti-CD47 antibodies (Biolegend, 323123), anti-integrin subunit αL antibodies (Abcam, ab52895), and Hoechst33342 at 4°C for 1 h in dark. After washing with pre-cooling PBS, cells were incubated with Alexa Fluor 488 conjugated Goat Anti-Rabbit IgG(H+L) (FMS-RBaf48801, Fcmacs) at 4°C for 1 h in dark. Then the cells were washed and prepared for detection under a laser confocal microscope (LSM800, Zeiss).

#### 5.7.2 Isolation of CD3+ T cells from wild-type or Cd47 knockout rats

Rat peripheral blood mononuclear cells (PBMCs) were first isolated from rat whole blood by density gradient equilibrium centrifugation with the use of a whole blood isolation kit (SolarBio, P8630) according to the instruction. Rat CD3+ T cells were isolated from PBMCs by the method of indirect magnetic beads. In brief, PBMCs were adjusted to 1×10^7^ to 10^8^ cells/mL and were incubated with an anti-CD3-PE antibody (miltenyi, 130-103-130) at 4°C for 30 min. Cells were washed with PBS containing 200 μg/mL EDTA-Na three times. Then cells were incubated with anti-PE microbeads (miltenyi, 130-048-801) at 4°C for 15 min. After washing the cells three times with the same washing buffer, cells were loaded onto MS Separation Columns (miltenyi, 130-042-201) for CD3+ T cell isolation.

#### 5.7.3 Activation of integrin α4β1 and αLβ2 by engagement of CD47

To confirm that CD47 engagement by 4N1K peptide can convert integrin α4β1 from a folding state to an extended state that is available for high-affinity ligand binding, an antibody (PS/2 clone, BE0071, BioXCell) that specifically binds to integrin α4β1 in an extended state was used in combination with a flow cytometry detection. In detail, 5×10^4^ human Jurkat cells were incubated with 2.5 μg/mL antibody in the presence or absence of 50 μM peptide 4N1K or the negative control peptide 4KGG. Human Jurkat cells incubated with an isotype antibody were used as an isotype control sample. FITC conjugated goat anti-rat IgG (H+L) (SA00003-11, ProteinTech) was used as a secondary antibody. The fluorescence signal of FITC labeled antibody binding to cells was detected by flow cytometry (CytoFlex, Beckman Coulter). Total expression of integrin α4β1 on Jurkat cells was confirmed with the flow cytometry method with the use of FITC conjugated mouse anti-human integrin α4β1 monoclonal antibody (304315, Biolegend).

The Extended form of integrin αLβ2 on Jurkat cells was detected with a similar flow cytometry method with the use of an anti-active form integrin αLβ2 antibody (FITC conjugated anti-human LFA-1 antibody, 363415, Biolegend). Human Jurkat cells incubated with an isotype antibody were used as an isotype control sample. Total expression of integrin αLβ2 on Jurkat cells was confirmed with the flow cytometry method with the use of a rabbit anti-human integrin αLβ2 monoclonal antibody (ab52895, abcam) and a secondary antibody (Alexa Fluor 488 conjugated Goat Anti-Rabbit IgG(H+L), FMS-RBaf48801, Fcmacs).

This detection was also performed with CD3+ T cells isolated from wild-type or *Cd47* knockout rats. 5×10^4^ CD3+ T cells from wild-type or *Cd47* knockout rats were incubated with 2.5 μg/mL antibody (PS/2 clone, BE0071, BioXCell) in the presence or absence of 50 μM peptide 4N1K or the negative control peptide 4KGG. Rat T cells incubated with an isotype antibody were used as an isotype control sample. A FITC conjugated goat anti-rat IgG (H+L) (SA00003-11, ProteinTech) was used as a secondary antibody. The fluorescence signal of the FITC-labeled antibody binding to cells was detected by flow cytometry (CytoFlex, Beckman Coulter). Total expression of integrin α4β1 on wild-type rat CD3+ T cells was confirmed with a flow cytometry method with the use of a rabbit anti-rat integrin α4β1 monoclonal antibody (8440T, Cell Signaling Technology) and Alexa Fluor 488 conjugated Goat Anti-Rabbit IgG(H+L) (FMS-RBaf48801, Fcmacs).

#### 5.7.4 Cell binding assays

For binding of human Jurkat cells to immobilized human VCAM-1 (**Figure 5E**), 96 well plate was coated with human VCAM-1 (4 μg/well) at 4°C overnight. A total of 5×10^4^ human Jurkat cells per well were added to the wells in the presence or absence of 50 μM peptide 4N1K or the negative control peptide 4KGG. After incubation at 37°C for 2 hours, unbound cells were carefully removed and the bound cells were stained with crystal violet solution. Bound cells were observed under a digital microscope (CKX41, Olympus, Japan).

For binding of rat CD3+ T cells to immobilized rat VCAM-1, 96 well plate was coated with rat VCAM-1 (4 μg/well) at 4°C overnight. A total of 5×10^4^ rat CD3+ T cells were added to each well in the presence or absence of 50 μM peptide 4N1K or the negative control peptide 4KGG. After incubation at 37°C for 2 hours, unbound cells were carefully removed and the bound cells were stained with crystal violet solution. Bound cells were observed under a digital microscope (CKX41, Olympus, Japan). First, CD3+ T cells from wild-type or *Cd47* knockout rats were added to the wells, and three experimental conditions were included: a control group without any peptide, a 4N1K group, and a 4KGG group (**Supplementary Figures 6A, B**). Furthermore, another three binding experiments were performed using CD3+ T cells from wild-type rats, each with three experimental conditions: a control group without any peptide, a 4N1K group, and a 4N1K peptide together with 50 nM PKA inhibitor (PKAI), 1 μg/mL PTX, or 40 μM Src kinase inhibitor (PP2) (**Supplementary Figures 7A–C**).

Binding of human Jurkat cells to immobilized human ICAM-1 was performed in a similar way with 96 well plate coated using human ICAM-1 (4 μg/well) (**Supplementary Figure 5E**).

#### 5.7.5 Cell migration assays

For migration of human Jurkat cells towards human VCAM-1 (**Figure 5F**), cells under various experimental conditions were resuspended in serum-free media. A total of 5×10^4^ cells per well were added to the upper compartment of the transwell chamber containing 5 μm pores (Millipore, MCMP24H48). The transwell chamber was previously coated with human VCAM-1 (4 μg/mL) at 4°C overnight. Cells in the upper chamber were incubated without or with 50 μM peptide 4N1K or the negative control peptide 4KGG. The chamber was placed into a 24-well plate with 600 μL of DMEM containing 10% FBS and 20 ng/mL SDF-1 (Sigma-Aldrich, SRP3276) and incubated for 8 h. After the removal of non-migrated cells, cells that had migrated through the filter were fixed with ethanol, stained with crystal violet solution, and counted with a digital microscope (CKX41, Olympus, Japan).

Migration of rat CD3+ T cells towards rat VCAM-1 was evaluated similarly to that for human Jurkat cells. A total of 5×10^4^ CD3+ T cells from wild-type or *Cd47* knockout rats were put in the upper chamber in the presence or absence of 50 μM peptide 4N1K or the negative control peptide 4KGG. The chamber was placed into a 24-well plate with 600 μL of DMEM containing 10% FBS and 20 ng/mL SDF-1 (Sigma-Aldrich, SPR3252) and was incubated for 8 h. Firstly, CD3+ T cells from wild-type or *Cd47* knockout rats were added to the upper chamber, and three experimental conditions were included: a control group without any peptide, a 4N1K group, and a 4KGG group (**Supplementary Figures 6C, D**). Furthermore, another experiment was performed using CD3+ T cells from wild-type rats, with six experimental conditions: a control group without any peptide, a 4N1K group, a 4KGG group, and a 4N1K peptide together with 50 nM PKA inhibitor (PKAI), 1 μg/mL PTX, or 40 μM Src kinase inhibitor (PP2) (**Supplementary Figure 7D**).

Migration of human Jurkat cells to immobilized ICAM-1 was performed in a similar way with transwell chambers coated using 4 μg/mL ICAM-1 (**Supplementary Figure 5F**).

### 5.8 Key intracellular signaling pathways for integrin α4β1 activation

#### 5.8.1 Phosphorylation of the intracellular part of integrin α4 subunit

Human Jurkat cells were incubated with 50 μM peptide 4N1K or 4KGG or 1 μg/mL natural TSP protein at 37°C for 2 h. Cells were lysed on ice in RIPA buffer (Cell Signaling Technology, Boston, MA). Quantified protein lysates were separated on an 8% gradient gel for western-blot analysis. After blocking of the membrane, total integrin subunit α and phosphorylated subunit α of integrin α4β1 were probed with an anti-integrin subunit α antibody (sc-376334, SANTA CRUZ) or an anti-phosphorylated subunit α antibody (sc-23943, SANTA CRUZ) overnight at 4°C, respectively. After washing, the membranes were incubated with secondary HRP-conjugated antibodies for 1 h at room temperature. The bands were detected using an ECL (enhanced chemiluminescence) kit (Tanon, China) and analyzed with ImageJ software.

#### 5.8.2 Effect of enzyme inhibitors on phosphorylation of the integrin α4 subunit

Human Jurkat cells were incubated with 50 μM peptide 4N1K in the presence or absence of 50 nM PKA inhibitor (PKAI), 1 μg/mL PTX, or 40 μM Src kinase inhibitor (PP2) at 37°C for 2 h. Phosphorylation of integrin α4 subunit of cells under various treatment conditions was detected with western-blot.

CD3+ T cells isolated from wild-type or *Cd47* knockout rats were incubated with 50 μM peptide 4N1K in the presence or absence of 50 nM PKA inhibitor (PKAI), 1 μg/mL PTX or 40 μM Src kinase inhibitor (PP2) at 37°C for 2 h. Phosphorylation of integrin α4 subunit of cells under various treatment conditions was detected with western-blot with the use of antibodies specific for rat integrin α4 subunit or phosphorylated α4 subunit.

#### 5.8.3 Detection of whole-cell cAMP and IP3 contents

Human Jurkat cells were incubated with or without 50 μM peptide 4N1K or 4KGG at 37°C for 2 h. Whole-cell cAMP and IP3 contents were analyzed with the use of human cAMP (mlbio, ml064278) and human IP3 (mlbio, ml060362) detection kits.

CD3+ T cells isolated from wild-type or *Cd47* knockout rats were incubated with or without 50 μM peptide 4N1K or 4KGG at 37°C for 2 h. Whole-cell cAMP and IP3 contents were analyzed with the use of rat cAMP (mlbio, ml002907) and rat IP3 (mlbio, ml059408) detection kits.

### 5.9 Statistical methods

The data was presented as the mean ± SEM or mean ± SD. All statistical tests were performed using SPSS19.0 (IBM) and GraphPad Prism 8.0 software. These results were performed by using a two-tailed Student’s t-test or one way ANOVA. P<0.05 was considered statistically significant.

## Data Availability

The original data of RNAseq analysis of synovial tissues of animals from different groups has been uploaded to NCBI SRA database with BioProject accession number of PRJNA1024082.
