# Supplementary material for: TSP-1-CD47-integrin α4β1 axis drives T cell infiltration and synovial inflammation in rheumatoid arthritis
Source: Front Immunol. 2025 Apr 16;16:1524304. doi: 10.3389/fimmu.2025.1524304 (PMC12040643; doi:10.3389/fimmu.2025.1524304)
Supplement: Supplementary file 1 [file DataSheet1.pdf]

Supplementary Figure 1

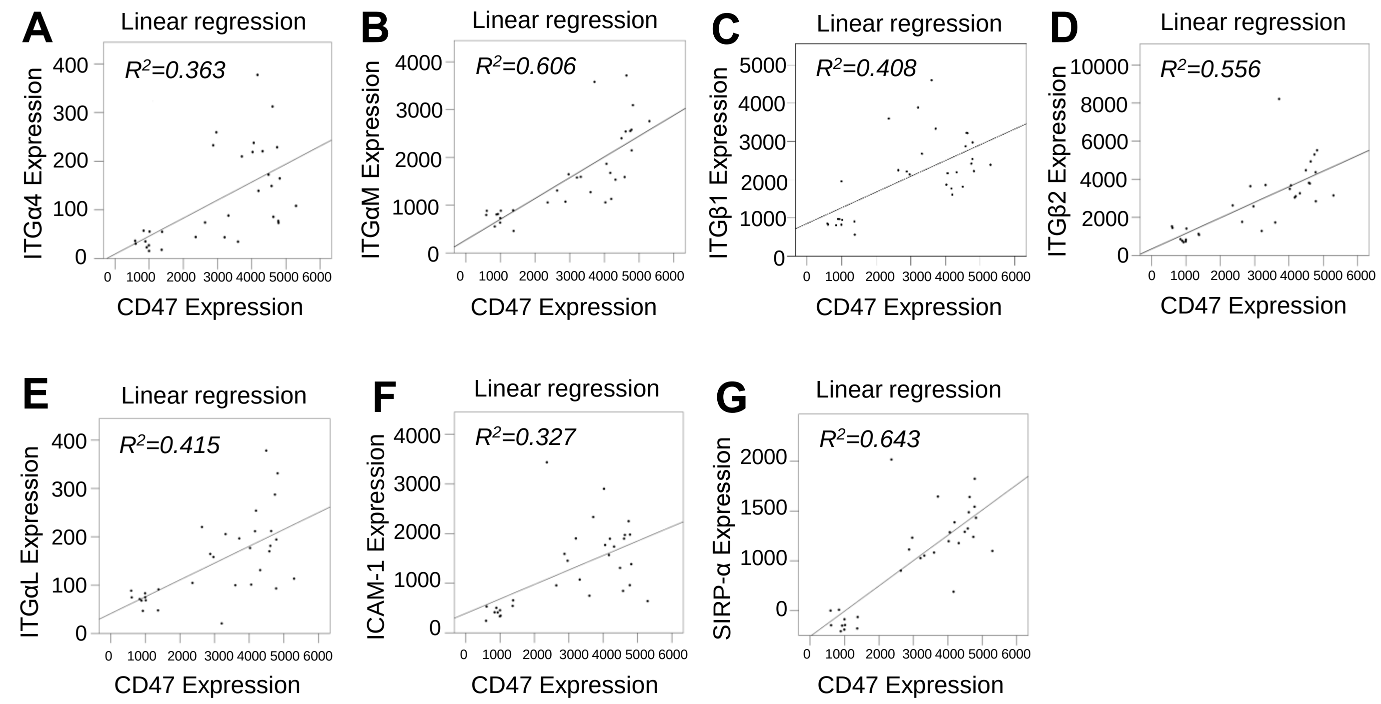

Supplementary Figure 1. Linear correlation of CD47 expression with the expression of several integrin subunits (Ŭ4, ŬM, ϐ1, ϐ2, ŬL), ICAM-1, and SIRP-Ŭ in synovial tissues. Data were based on published GEO profiles (NCBI/GEO/GSE 55457/77298/55235).

Supplementary Figure 2

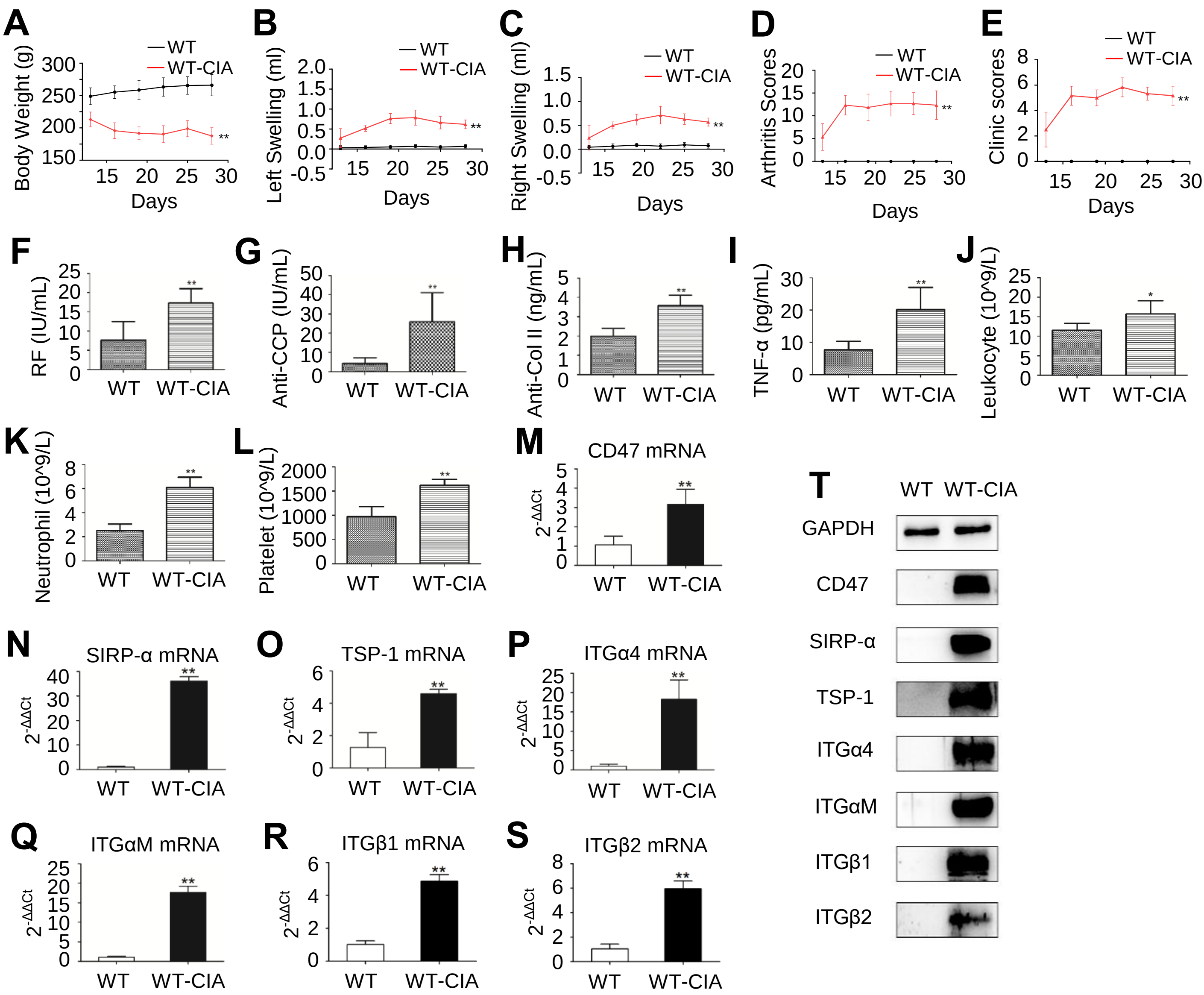

Supplementary Figure 2. Establishment of collagen-induced arthritis (CIA) in wild-type SD rats. Two groups of rats were included: the normal group (WT, the black line) and the model group (WT-CIA, the red line) (n=6). **A**-**E**. Change of body weight (**A**), swelling of hind left paws (**B**) and right paws (**C**), the arthritis score (**D**) and clinic score (**E**) of rats in both groups during the experiment. On day 28 rat blood was collected and the levels of molecules or cells were measured and compared which include rheumatoid factor (**F**), anti-CCP antibodies (**G**), anti-CII antibodies (**H**), TNF-α (**I**), total leukocytes (**J**), neutrophils (**K**) and platelets (**L**). For samples in **A** to **L**, n=6. Rat synovial tissue was collected on day 28 for qPCR analysis and western-blot analysis. **M** to **S**. Expression of CD47 (**M**), SIRP-α (**N**), TSP-1 (**O**), integrin subunit α4 (**P**), αM (**Q**), β1 (**R**), and β2 (**S**) on an mRNA level in rats of both groups were shown and compared (n=3, three replicates for detection of each molecule). Data show mean ± SD. $*P < 0.05$, $**P < 0.01$, $***P < 0.001$. Photo of WB (**T**) for the expression of CD47, SIRP-α, TSP-1, integrin subunit α4, αM, β1 and β2 in synovial tissue of rat hind paw was shown.

Supplementary Figure 3

A

```
              170         180         190        200
WT        AAAACTTTACCAGTGCAAAAATCTCGGTCTCAGACTTGCT
CD47-KO   AAAACTTTACCAGTGCAAAAATCTCGGTCTCAGACTTGCT

              210         220         230         240
WT        CAAAGGCATTGCCTCTTTGACAATGGATACCCATGAGGCT
CD47-KO   CA................................TGAGGCT

               250         260         270
WT        GTGGTGGGAAACTACACCTGTGAAGTGACTGAG
CD47-KO   GTGGTGGGAAACTACACCTGTGAAGTGACTGAG
```

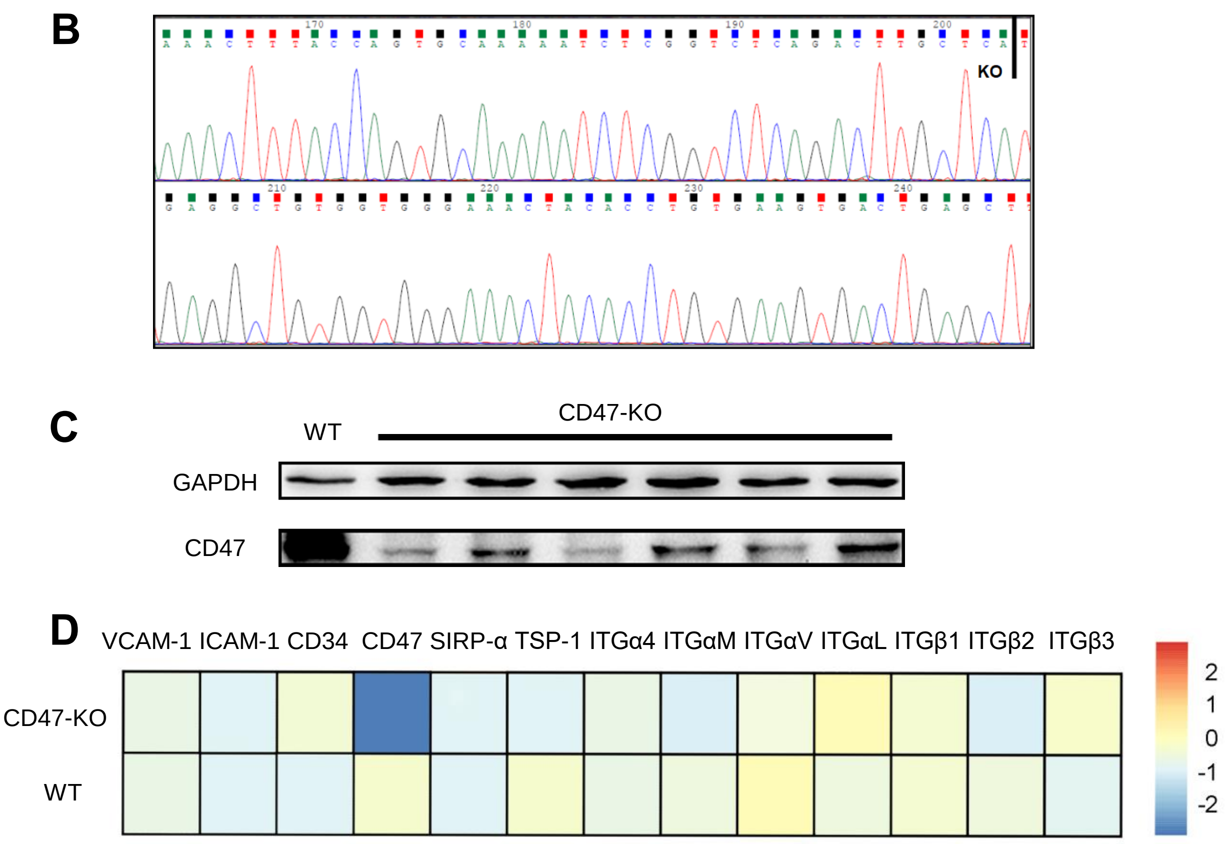

Supplementary Figure 3. Generation of CD47 knockout rats. CD47 knockout rats were generated by removal of 31 bp target site in exon 2 of CD47 gene with the CRISPER/ Cas9 technology. **A**. The removed 31 bp target site of exon 2 of the CD47 gene. **B**. Sequencing of target site after removal of the 31 bp. **C**. Expression of CD47 in homozygous CD47 knockout F2 rats on a protein level. **D**. Relative expression of several relevant proteins in a CD47 knockout rat and a wild-type rat. Relative quantitative analysis of protein expression in tail tissues of wild-type and CD47 knockout rats was performed with a TMT labeling and LC-MS/MS method.

Supplementary Figure 4

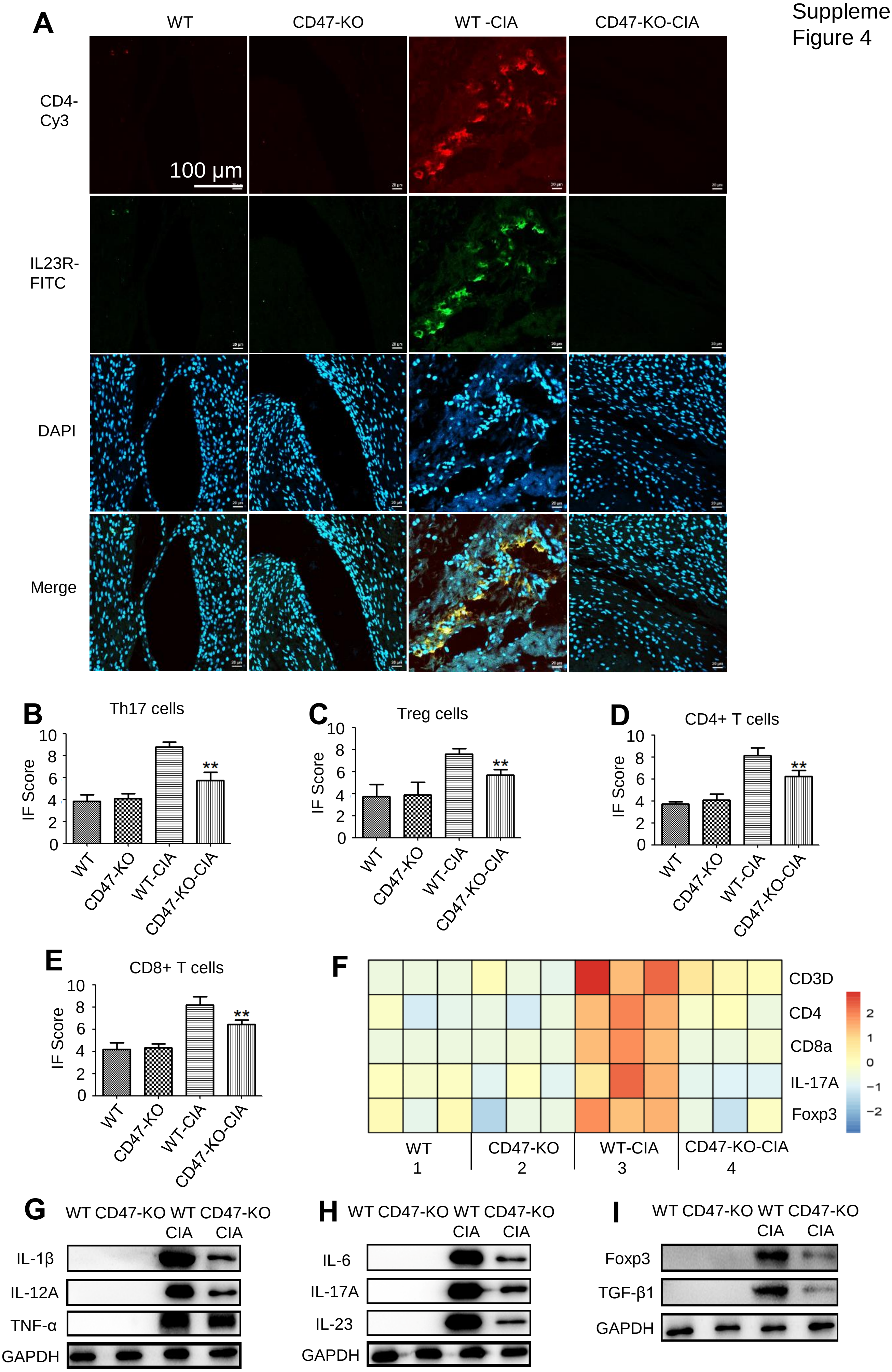

Supplementary Figure 4. Decreased numbers of T cell subgroups in the synovial membrane of CD47 knockout rat model group. Hind paw joints were taken from rats in the animal experiment as shown in Figure 2 on day 28 and used as samples for immunofluorescence staining analysis. **A**. Immunofluorescence staining of IL-23R (a marker for Th17) expression on hind paw sections was obtained at the end of the experiment for rats in the four experimental groups (400×). IL-23R molecule was labeled with FITC. CD4 molecule was labeled with Cy3. DAPI was used to stain the cell nucleus. The merge of the IL-23R signal and the CD4 signal showed the presence of Th17 cells. **B**. Fluorescence signal analysis confirmed significantly fewer Th17 cells in the joints of CD47 knockout rat model group compared with wild-type rat model group (n=3, three photos for each experimental group). Data show means ± SD. *$p < 0.05$, **$p < 0.01$ versus the wild-type rat model group. **C-E**. Fluorescence signal analysis confirmed significantly fewer Tregs, CD4+T cells and CD8 + T cells in the joints of CD47 knockout rat model group compared with wild-type rat model group (n=3, three photos for each experimental group). Data show mean ± SD. *$p < 0.05$, **$p < 0.01$ versus the wild-type rat model group. For CD25 staining (a marker for Tregs), the merge of the CD25 signal (FITC labeling) and the CD4 signal (Cy3 labeling) showed the presence of Tregs. For CD4 staining, the merge of the CD4 signal (FITC labeling) and the CD3 signal (Cy3 labeling) showed the presence of CD4+ T cells. For CD8 staining, the merge of CD8 signal (FITC labeling) and CD3 signal (Cy3 labeling) showed the presence of CD8+ T cells. DAPI was used to stain the cell nucleus. **F**. Differential expression of markers for T cell subgroups (CD3D, CD4, CD8a, IL-17A and FoxP3) in synovial membranes of rats in the four groups by RNAseq analysis (n=3, samples of three animals for each group). **G-I**. Western-blot analysis of relevant expression molecules related to CD4+T cells (IL-1β, IL-12A, and TNF-α), Th17 cells (IL-6, IL-17A, and IL-23), and Treg cells (FoxP3 and TGF-β1) in synovial membranes of rats in the four groups.

Supplementary Figure 5

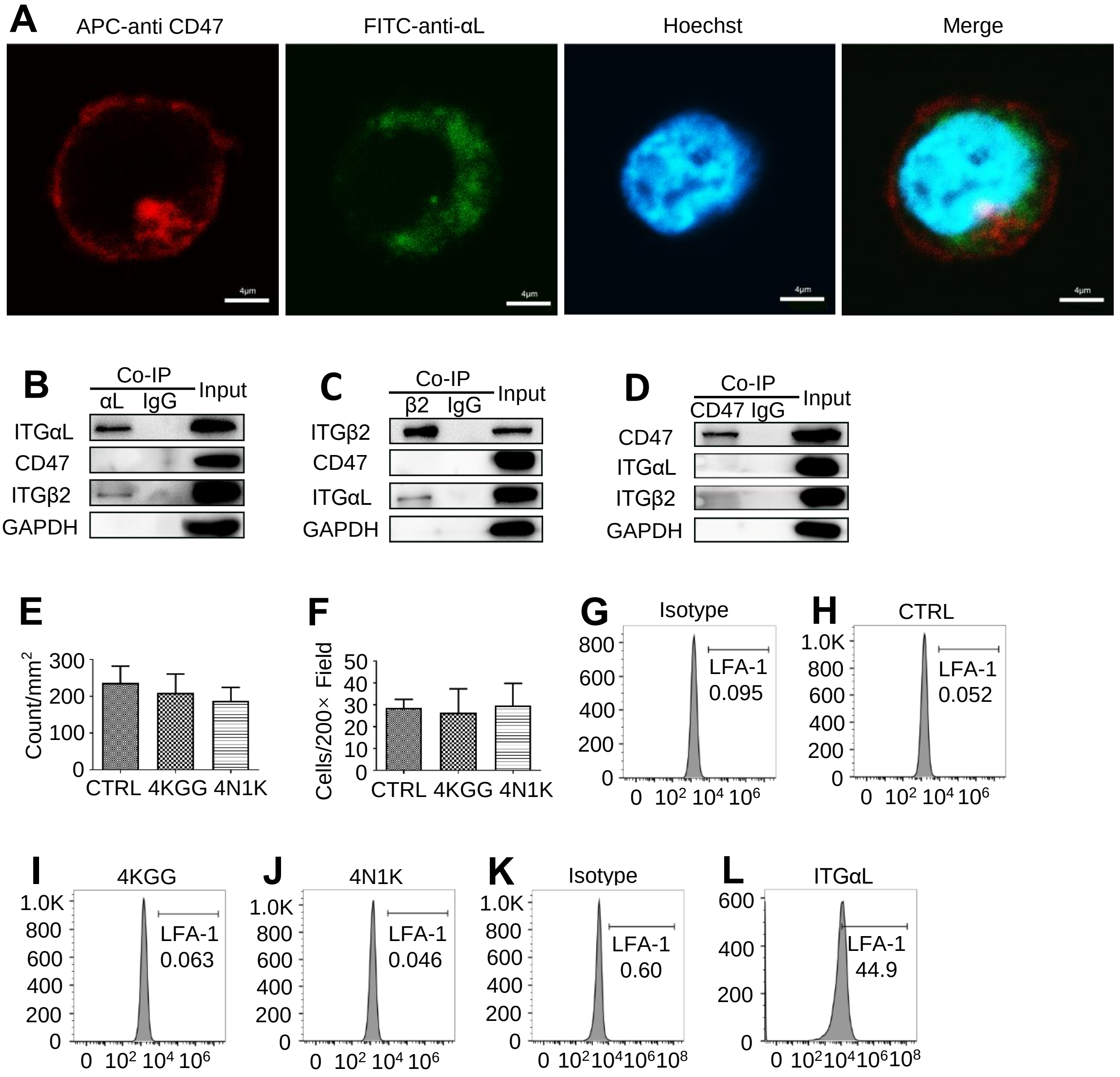

Supplementary Figure 5. Peptide 4N1K engagement on CD47 had no effect on integrin αLβ2 activation and function on human Jurkat cells. **A**. CD47, αL integrin, and cell nucleus were probed and stained by an APC conjugated anti-CD47 antibody, a FITC conjugated anti-αL antibody or Hoechst staining and no merge signal appeared which showed that CD47 and αL integrin had no direct interaction on Jurkat cell surface (400×). **B**-**D**. Western-blot analysis of the bands for CD47, αL, and β2 integrin subunit after immunoprecipitation with an anti-αL (**B**), anti-β2 (**C**), or anti-CD47 (**D**) antibody. Cell lysate which was negatively labeled in the middle was precipitated with an isotype control antibody. The whole-cell lysate on the right was named input. **E**. Peptide 4N1K treatment did not affect the adhesion of human Jurkat cells to immobilized ICAM-1 (n=3, three replicate samples for each experimental condition). Cells in the control sample were not incubated with peptides. **F**. Peptide 4N1K treatment did not affect the migration of human Jurkat cells to immobilized ICAM-1 with SDF-1 as a chemoattractant (n=3, three replicate samples for each experimental condition). For **E** and **F**, data show mean ± SEM. $*P < 0.05$, $**P < 0.01$. **G**-**J**. Peptide 4N1K treatment did not increase the percentage of "extended form" integrin αLβ2. Cells were treated with peptide 4KGG (**I**), 4N1K (**J**), or without peptide treatment (**H**) and then were probed with a FITC conjugated anti-active form integrin αLβ2 antibody. Human Jurkat cells incubated with an isotype antibody were used as an isotype control sample (**G**). Total expression of integrin αLβ2 on Jurkat cells was confirmed with the use of a rabbit anti-human integrin αLβ2 monoclonal antibody and an Alexa Fluor 488 conjugated secondary antibody (**L**). Jurkat cells incubated with an isotype antibody were used as an isotype control sample (**K**).

Supplementary Figure 6

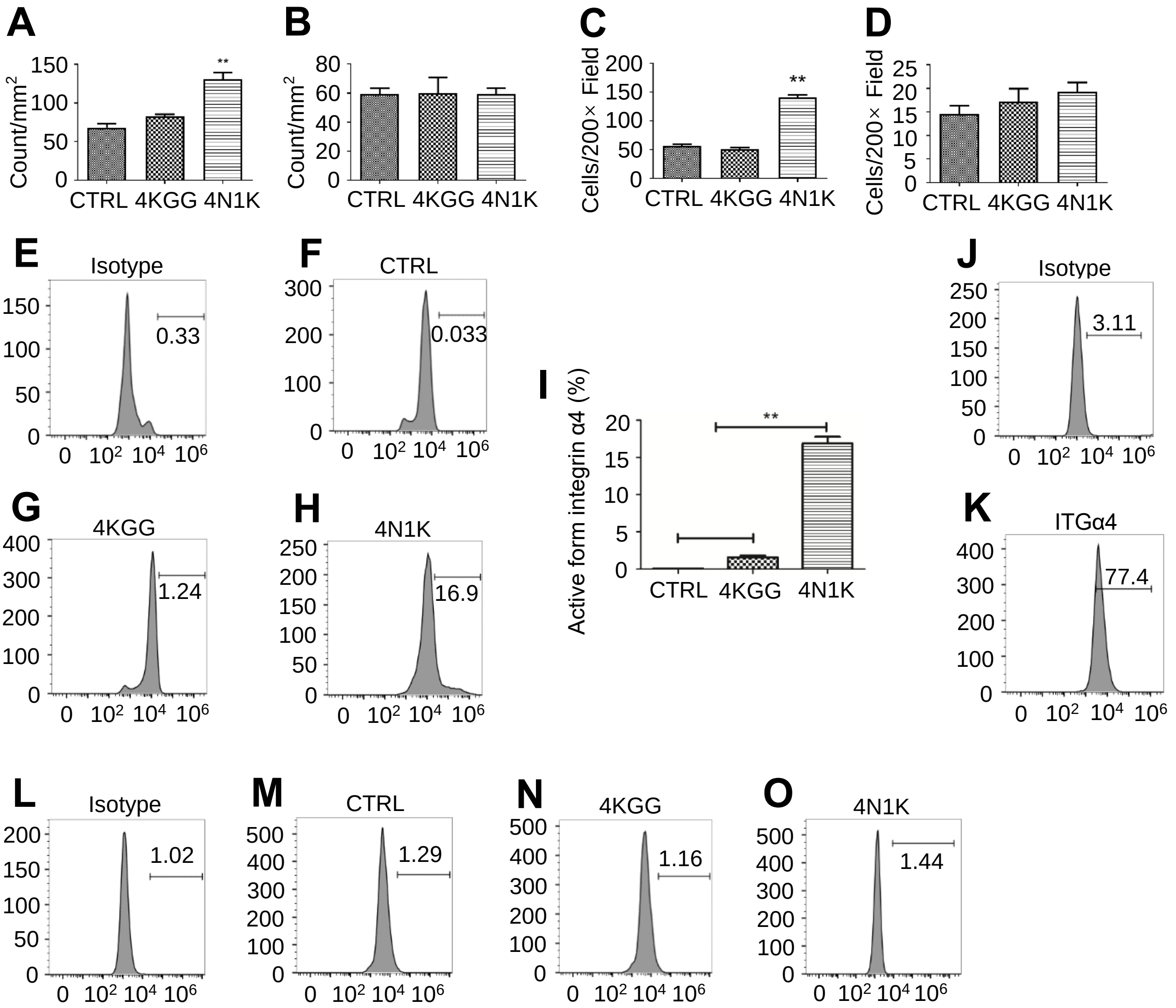

Supplementary Figure 6. Effect of peptide 4N1K treatment on adhesion, migration, and integrin α4β1 activation of rat CD3+ T cells. Effect of peptide 4N1K and 4KGG on adhesion of wild-type (**A**) or CD47 knockout (**B**) rat CD3+ T cells to immobilized VCAM-1 (n=3, three replicate samples for each experimental condition). Cells in the control sample were not incubated with peptides. Transwell analysis of the effect of peptide 4N1K and 4KGG on the migration of wild-type (**C**) or CD47 knockout (**D**) rat CD3+ T cells to immobilized VCAM-1 with SDF-1 as a chemoattractant (n=3, three replicate samples for each experimental condition). **E-H**. Flowcytometry assay of percent increase of "extended form" integrin α4β1. CD3+ T cells isolated from wild-type rats were treated with peptide 4KGG (**G**), 4N1K (**H**), or without peptide treatment (**F**) and then were probed with an anti-active form integrin α4β1 antibody, and thereafter a FITC-labeled secondary antibody. Cells incubated with an isotype antibody were used as an isotype control sample (**E**). **I**. Comparison of up-regulation of integrin α4β1 active form under various treatment conditions (n=3, three detections for the same experimental condition). All of the data show mean ± SEM. $*P < 0.05$, $**P < 0.01$, $***P < 0.001$. Total expression of integrin α4β1 on wild-type rat CD3+ T cells was confirmed with the use of a rabbit anti-human integrin α4β1 monoclonal antibody and an Alexa Fluor 488 conjugated secondary antibody (**K**). Jurkat cells incubated with an isotype antibody were used as an isotype control sample (**J**). **L-O**. CD3 + T cells isolated from CD47 knockout rats were treated with peptide 4KGG (**N**), 4N1K (**O**), or without peptide treatment (**M**) and then were probed with an anti-active form integrin α4β1 antibody, and thereafter a FITC-labeled secondary antibody. Cells incubated with an isotype antibody were used as an isotype control sample (**L**).

Supplementary Figure 7

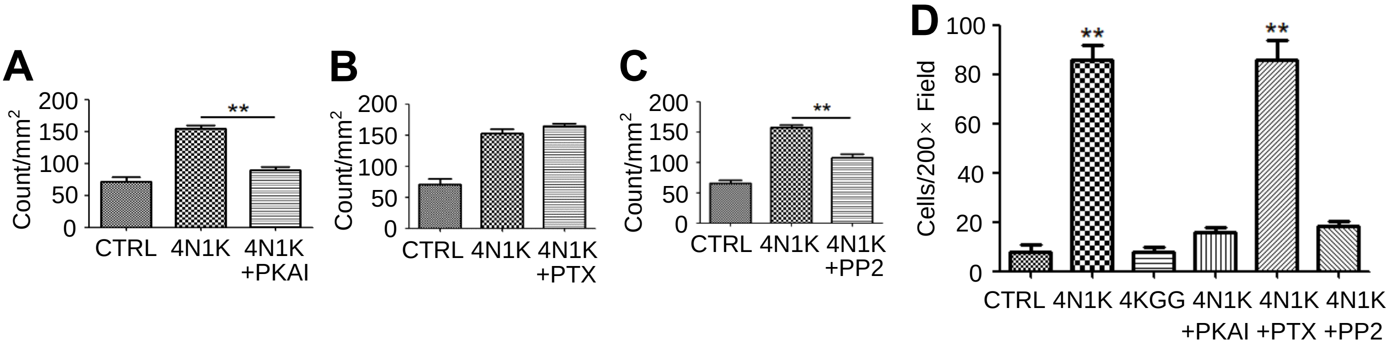

Supplementary Figure 7. In vitro effect of PKAI, PTX, or PP2 incubation on wild-type rat T cell adhesion and migration. Effect of PKAI (**A**), PTX (**B**), or PP2 (**C**) on peptide 4N1K stimulation of cell adhesion to immobilized VCAM-1 (n=3, three replicate samples for each experimental condition). **D**. Effect of PKAI, PTX, or PP2 on peptide 4N1K stimulation of cell migration towards immobilized VCAM-1 with SDF-1 as a chemoattractant (n=3, three replicate samples for each experimental condition). All of the data show mean ± SD. *P < 0.05, **P < 0.01, ***P < 0.001.
